# Mapping social accountability actors and networks and their roles in water, sanitation and hygiene (WASH) in childcare centres within Nairobi’s informal settlements: A governance diaries approach

**DOI:** 10.1371/journal.pone.0275491

**Published:** 2022-11-15

**Authors:** Ivy Chumo, Caroline Kabaria, Penelope A. Phillips-Howard, Sheillah Simiyu, Helen Elsey, Blessing Mberu

**Affiliations:** 1 African Population and Health Research Center (APHRC), Nairobi, Kenya; 2 Department of Clinical Sciences, Liverpool School of Tropical Medicine, Liverpool, United Kingdom; 3 University of York and Hull York Medical School, York, United Kingdom; Georgia Institute of Technology, UNITED STATES

## Abstract

**Introduction:**

Despite many institutions gaining access to improved water sanitation and hygiene (WASH) services, childcare centres in informal settlements have low access and poor condition of WASH services. It is imperative to understand how existing actors and social networks operate in the WASH sector in childcare centres in Nairobi’s informal settlements.

**Objective:**

To empirically map and understand how different actors within informal settlements influence the provision of adequate and quality water, sanitation and hygiene services within childcare centres in Nairobi’s informal settlements.

**Methods:**

This was a qualitative study. We conducted an ethnographic study using governance diaries with 24 participants from Korogocho and Viwandani informal settlements in Nairobi, Kenya. The governance diaries approach involved conducting bi-weekly governance in-depth interviews (IDIs) with study participants for 4 months, complemented with observations, reflections, participant diaries and informal discussions. We used a framework analysis which is partly deductive, informed by the governance framework and stakeholder framework.

**Results:**

Social accountability actors were individuals or groups involved in WASH service provision in childcare centres. The actors included both key actors (actors who are primary to meeting the day-to-day WASH service needs of children) and non-key actors (actors operating in the WASH sector but not always present for day-to-day provision in childcare centres). The key actors were unanimously identified as childcare centre owners/teachers and parents/guardians as they had a more direct role in the provision of WASH services in childcare centres. The actors had direct, possible or desired networks, with the direct networks portrayed more by the parents and childcare centre owners, whose roles included acting as a voice and responding to the WASH service needs of children as it relates to access and quality. Centre owners had more power/authority over WASH services for children in childcare centres than the parents. Key actors derived power by their discretion depending on whether a decision was beneficial to children or not. Lastly, the interest of key actors were diverse ranging from income generation, access to WASH services by children, compliance with government regulations, and promotion of child health, to the prevention of the spread of diseases.

**Conclusion:**

Our study highlights that parents and childcare owners play an important role in WASH service provision. While service providers and other players may be statutorily given primary responsibilities for WASH provision, and more visible in official standing, among study participants they are not seen as primary actors but secondary players with ancillary responsibilities. We conclude that WASH service provision in child care centres may be realised when key actors have a voice and work within networks to demand WASH services from desired networks including the government. We also conclude that developing more direct networks and converting desired and potential networks into direct networks in WASH service provision is critical for the success of WASH service delivery. Lastly, actors in WASH services in childcare centres may need to collaborate in identifying potential avenues for strengthening existing networks that enhance access and quality of WASH services in childcare centres.

## 1. Introduction

Informal settlements are growing as more women work outside home for long hours in the city, and childcare centres have mushroomed to fill the childcare vacuum [1,2]. Providing adequate water sanitation and hygiene (WASH) services in childcare settings in informal settlements is challenging, yet, it is important for child health and development [2]. Globally, about 15,000 children under five die from infections that could have been prevented with low-cost interventions, such as water, sanitation and hygiene service delivery [3,4]. Children have a right to basic facilities such as access to toilets, safe drinking water, and hygiene [5]. Physiologically, if conditions for quality water, sanitation and hygiene are created, children learn better and can take concepts and practices on sanitation and hygiene back to their families [2,6]. WASH is essential for progress toward current global priorities in the sustainable development goals (SDG) [7], particularly, universal high-quality pre-primary education in a safe, effective, and inclusive environment (SDG 4); and universal access to clean water and sanitation (SDG 6) [8]. Incorporating quality WASH in childcare centres enhance programme quality [9–11]; improves young children’s well-being [12]; and establishes positive WASH-related attitudes, skills and behaviours before school-entry age for sustainable management of WASH services [2,13]. Despite significant investment over the past decades, and many people gaining new access to quality WASH services [12], there are gaps in governance of WASH service delivery in childcare centres in informal settlements, portrayed in weak social accountability structures (actors and networks) [14,15].

Informal settlements (i.e unplanned sites that are not compliant with authorized regulations) are characterised by weak social accountability [16]. Social accountability is a component of governance that encompasses citizens’ voices, policymakers’ enforceability and service providers’ answerability [17,18]. Social accountability for WASH services in Nairobi County are decentralised, and capacities for demanding the services may be weak with limited resources. Therefore, central or local governments may forgo service provision and residents end up relying on the private and informal sectors for the provision of WASH services [19,20]. Under such circumstances, there is a change in the set of relationships between the state and its citizens, and users may find themselves unable to hold the existing providers or agents to account, so leaving communities to find ways of fulfilling their own ‘WASH’ needs, as well as some Non-Governmental Organisations (NGOs) coming in to fill the gaps in service provision.

**Fig 1:**
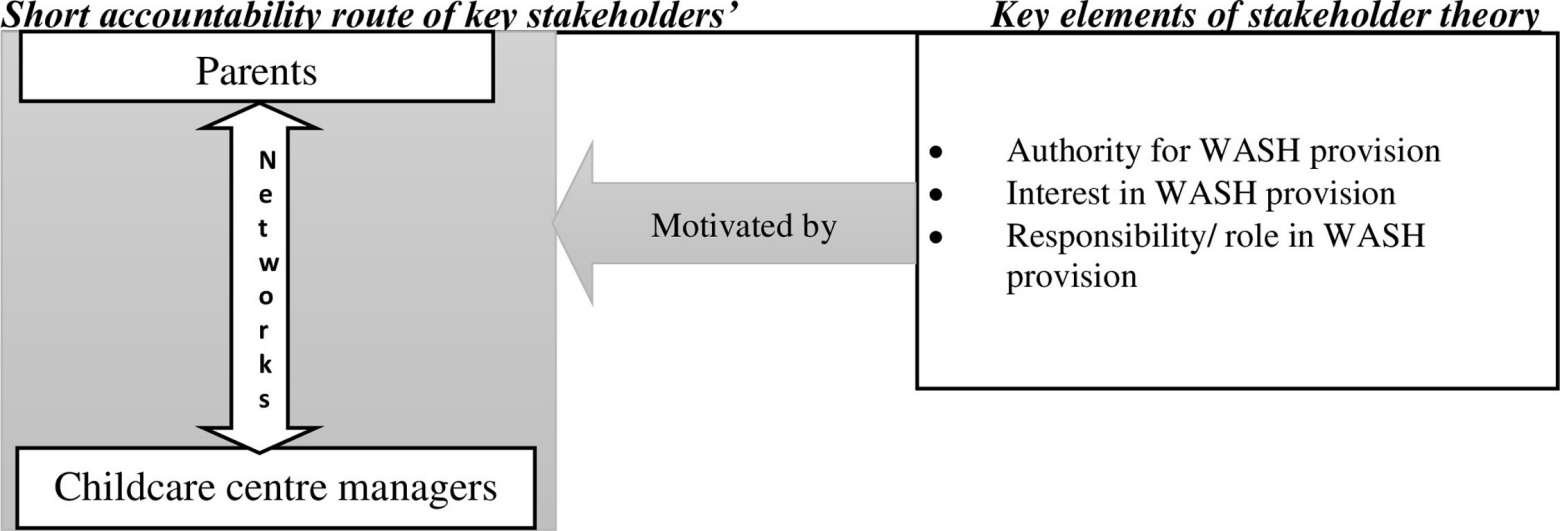
Conceptual framework.

Access to WASH services in schools in Nairobi’s informal settlements is a key challenge affecting child health [19]. In childcare centres, most of the water sources are water vendors with public taps, sanitation points are portable toilets (potties), disposed of in pit latrines, and hand washing stations/facilities are less documented in childcare centres [20,21]. Water is paid for by users who could be business owners, childcare owners, or homeowners, and there is more access to WASH services in structures near main paths/roads than those further from main paths/roads [20,22]. In the childcare centres, like in other settings within informal settlements the provision of WASH services to a large extent, relies on local social accountability structures that are informal (i.e unstructured with loose ties), hence undervalued and unrecognized by formal actors (i.e actors with defined and structured guidelines) [23–25]. Most parents and childcare managers in informal settlements do not operate using centralised rules and guidelines but rely on contractual/unplanned arrangements for WASH service delivery [26]. A focus on contractual relationships between the parents and childcare managers in the WASH sector in childcare centres in informal settlements can lead to an increased WASH service delivery in a network of actors [14,17], yet it is underscored and rarely documented. Therefore, we empirically mapped actors and networks; roles; interests; and authority, depicted in a conceptual framework (Fig 1). We were guided by five key questions: a) who are the actors in WASH in childcare centres? b) what networks exist for the actors in WASH service provision in childcare centres? c) what is the role of actors in WASH in childcare centres? d) what interests do the actors have? e) where do the actors get their authority? By focusing on the five questions, this paper covers an important gap in the social accountability in WASH sector in childcare centres and contributes to thinking about interventions to improve access and quality of WASH services at large.

### Theoretical background and conceptual framework

We grounded our work on the World Bank accountability model and Stakeholder Theory. The World Bank accountability model has a long and short route to accountability [27–29]. The long route to accountability has an indirect relationship between service users and service providers via elected politicians/government officials, which is not common or nearly absent in informal settlements. This study was anchored on the short route to accountability with a direct relationship between users and service providers through the exercise of client power, as users individually and collectively participate and supervise service delivery directly [28]. On the other hand, stakeholder theory states that managers in organizations have a network of relationships that includes employees and partners. The theory focuses on the need for authority/managerial decision making, interests of stakeholders on intrinsic value, and further stresses on the role of actors [30,31]. Within our context, key stakeholders for WASH service provision in childcare centres are parents and childcare managers, as they influence the WASH services. The theory emphasizes the importance of network of actors, actors’ role, as well as their interests and power/ authority concerning WASH service provision in childcare centres. The short accountability model and stakeholder theory are applicable in this study as we present findings on actors and networks, authority, interests and responsibility of parents and childcare managers as summarized in Fig 1.

The conceptual framework describes how two groups of key actors/stakeholders, i.e (1) right holders/parents and (2) duty bearers/teachers or centre managers, interact with one another through direct networks. We build on the premise that parents and childcare centre managers have direct networks, which are motivated by key elements of authority, interest and responsibility for WASH service provision in childcare centres. The elements are interrelated, such that one aspect influence a potential to achieve success in other aspects [30,31]. For example, the role of advocating for access to WASH service delivery in childcare centres is not likely to be sustainable without some degree of interest from actors. The framework potentially promises to reduce working in silos as it allows stakeholders to operate in networks in the absence of formal actors [32].

## 2. Methods

The study is reported per a set of standardized criteria for reporting qualitative research (COREQ) [33].

### Study design

This was a qualitative study using the governance diaries method. Governance diaries is an ethnographic approach using more than one method of data collection and where participants make regular records of their daily activities and experiences [34–36]. Governance diaries are typically used in contexts where there is a need to explore the depth of everyday life, as time allows researchers to spend longer periods in the field for exploration [34,37]. For this study, governance diaries included in-depth interviews (IDIs), which were informed by participant diaries, informal discussions, participant observations and reflections.

### Study setting

**Fig 2:**
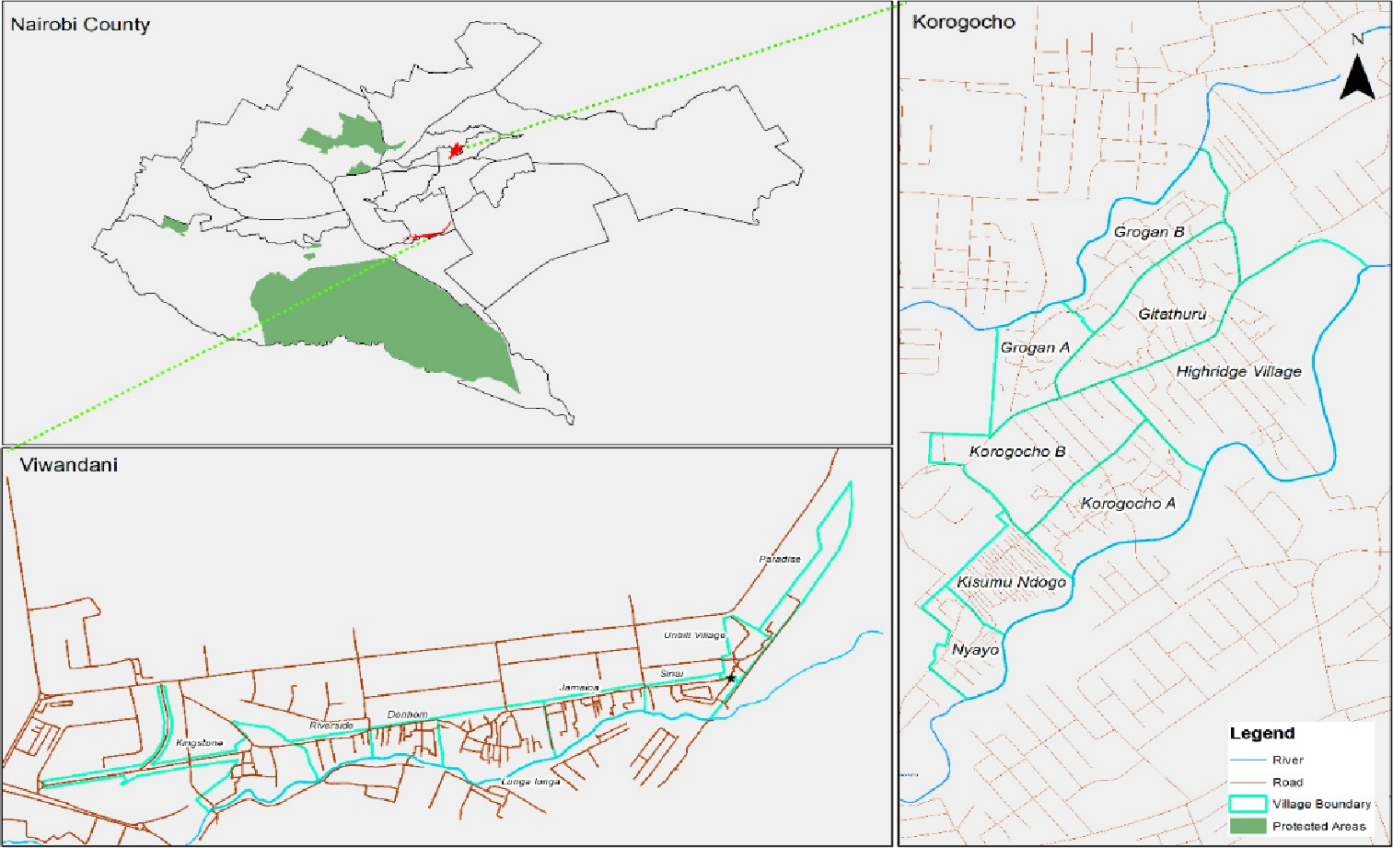
Study sites.

The study was conducted in Korogocho and Viwandani informal settlements in Nairobi, in the areas covered by Nairobi Urban Health and Demographic Surveillance System (NUHDSS) initiated in 2002 by the African Population and Health Research Center (APHRC) [38]. Korogocho has a stable and settled population and residents have lived in the area for many years [39], while Viwandani is located next to an industrial area with many highly mobile residents who work or seek jobs in the industrial area [39]. Each of the informal settlements has 8 subdivisions/units/villages, which acted as a guide during the selection of study participants (Fig 2). There are approximately 50 and 60 childcare centres in Korogocho and Viwandani respectively with poor or no access to WASH services [20]. Close to 70% of childcare centres have between 10 and 25 children, with many owned individually, few owned by faith-based organizations and very few are centre-based, operating mostly during the day (between 6:00 am and 5:00 pm) [1,11,40].

### Target population

The population of interest was childcare managers and parents.

### Sampling and sample size

We sampled 24 participants comprising of 4 childcare centre owners catering to children under-five years and 8 parents of children under-five years attending the childcare centres in each of the 2 study sites. For each of the centres where the centre managers were purposively recruited, two parents with children in each of the centres were purposively selected (Table 1). We purposively selected parents/guardians who were key for child expenses in the family. The 8 parents/guardians represented each of the 8 villages/units for each study site and had an under-five child attending childcare centres. The criteria for the selection of childcare centres were based on centre managers in centres that had been in existence longer than before the Covid-19 outbreak starting March 2020 and served children <5 years old. The selection criteria enabled the researchers to engage a study group with long tenure, representing all the units/villages in the study sites and with experience in WASH service delivery and social accountability in childcare centres.

**Table 1:** Sample size and demographic information.

	Korogocho	Viwandani
**Childcare managers**		
Sex	4 Female	4 Female
Education level	Primary = 1 Secondary = 2 Tertiary = 1	Primary = 1 Secondary = 1 Tertiary = 2
**Sub-total**	**4**	**4**
**Parents**	8 (each from 1 of the 8 villages/units)	8 (each from 1 of the 8 villages/units)
Sex	6 Female & 2 Male	6 Female & 2 Male
Educational Level	Primary = 3 Secondary = 4 Tertiary = 1	Primary = 2 Secondary = 3 Tertiary = 3
**Sub-total**	**8**	**8**
**Total**	**12**	**12**

### Data collection process

**Fig 3:**
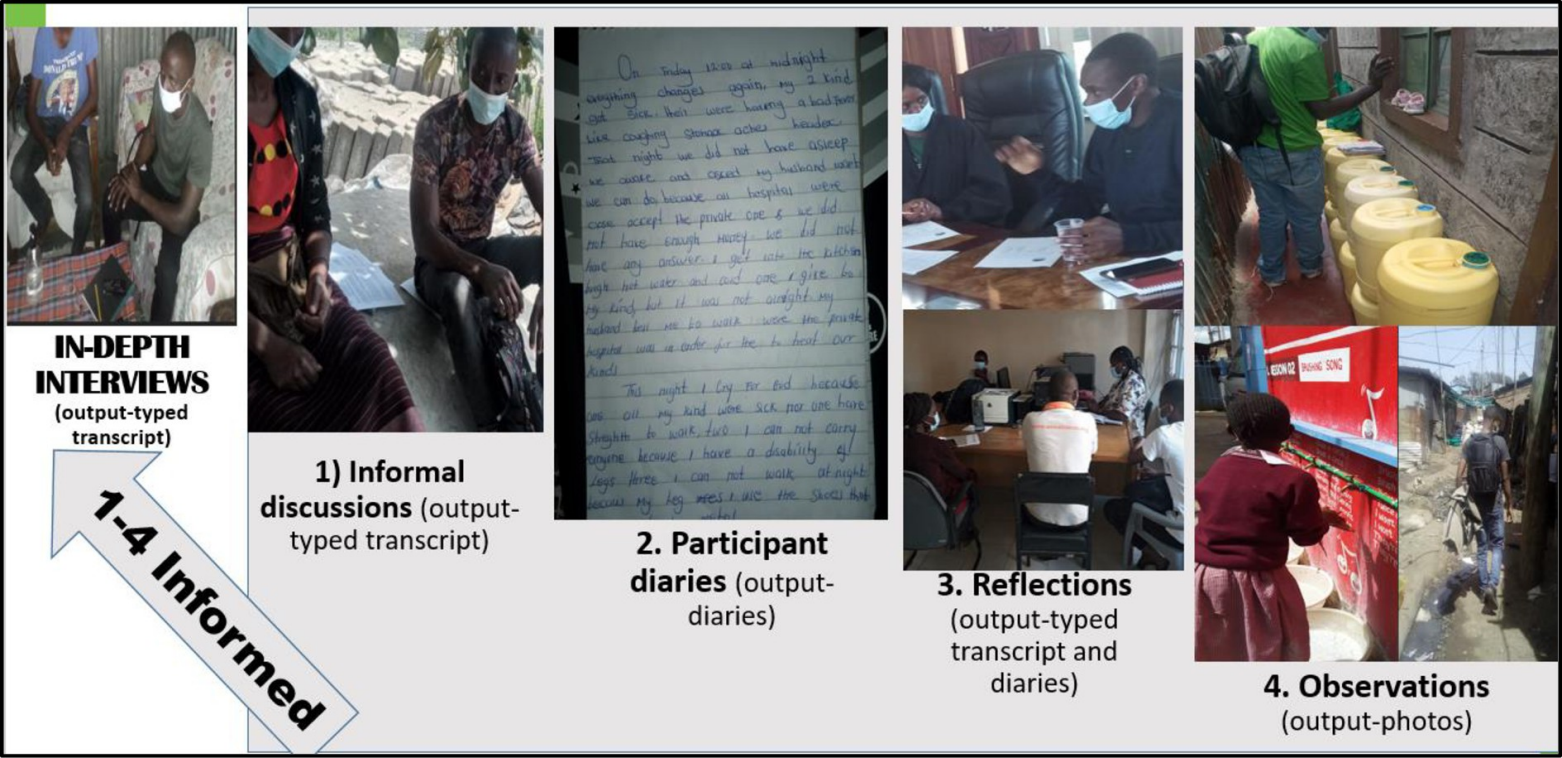
Data collection process.

Community Advisory Committees (CACs) (individuals selected by the community to represent and act as a liaison between the researchers and the community), co-researchers (community members recruited as research assistants because they have a better understanding of the context and with closer rapport with respondents) and the researchers worked together in the recruitment of study participants. We used governance diaries to collect data from January to April 2021 with questions related to actors, networks, roles, interests and authority of WASH actors in childcare centres. Diaries were implemented through In Depth Interviews (IDIs), participant observation, participant diaries, reflection and informal discussions. IDIs were the dominant method and were informed by the other methods. Below is the description of the data collection methods;

*Informal discussions*: an informal conversation was carried out between the participants and the researchers to find out key insights and to create rapport with the study participants before the IDIs;

*Reflections*: Reflective discussions were held between pairs of co-researchers on a daily basis, among the whole group of co-researchers on a weekly basis, and between researchers and co-researchers every two weeks, to understand the outcome and determine emerging themes and gaps to be probed during subsequent IDIs and routine observations;

*Observations*: These included observation by the co-researchers, which allowed for a holistic awareness of events as they unfold and as such, a more comprehensive understanding of what matters to respondents. The co-researchers observed the environment related to the study subject of water, sanitation and hygiene facilities and practices in childcare centres. This observation resulted in photos and insights on what to probe in the IDIs. The co-researcher whose role was to be an observer did the observations before, during, and after the IDIs to complement the discussions recorded and to inform and complement participant diaries, reflective discussions and IDIs. Reflective discussions informed the content and concepts for observations;

*Participant diaries*: We provided the study participants with guidelines pasted on the front of the diary. Each participant would write on a daily basis activities related to actors, networks, roles, authority and interest in WASH services in childcare centres, without writing their names. Co-researchers would call participants and conduct impromptu visits to remind participants about diary writing activities;

*IDIs*: We used guides with questions on actors, networks, roles, interests and authority of actors in WASH services delivery in childcare centres. IDIs for subsequent visits on the same questions were adapted based on observations, reflections, informal discussion and participant diaries (Fig 3). In-depth conversation/discussion between the research assistant and the study participants were administered in pairs of 2 co-researchers; with one moderating the discussion and the second acting as an observer, note taker and facilitating the recording of the conversations. We reached saturation in the IDIs during the sixth visit when we approached the fourth month.

The outputs from informal discussions, observations, participant diaries and reflections informed and enhanced robust probing during IDIs. For example, if the co-researchers observed some water pipe bursts at the childcare centre, during the IDIs the co-researcher would probe more about the water pipes. The multi-pronged ethnographic data collection processes are summarized in Fig 3.

Research assistants/co-researchers received training for 6 days on the aims of the study, data collection process, data collection tools, and research ethics. We piloted the study tools with one centre manager and 2 parents in each of the study sites, followed by a debriefing to assess if the study approach and study tools were well understood by both research assistants and study participants. The pilot exercise also enabled us to adjust the translated guides to concepts understood by the study participants and to estimate the time an interview would take. We excluded participants in the pilot from the main study. During fieldwork, researchers accompanied the co-researchers to assess data quality through spot-checks. Team debriefing sessions were held at the end of each working day to assess progress and ensure quality data collection processes. Researchers and co-researcher debriefings were conducted weekly for cross-learning, while research assistant reflective sessions were held after every round of the six visits to reflect on the previous round of data collection and to strengthen research skills for the next round of visits.

All the participants selected declared willingness to be involved in our study for 4 months at the onset of the study and no participant dropped out from the interviews.

### Data management

Recorded audios from IDIs, reflections and informal discussions were translated and transcribed from Swahili to English and saved as individual Microsoft Word documents. Outputs were assigned number codes to prepare for analysis and to ensure confidentiality. Thereafter, transcripts were imported into NVivo 12 software (QSR International, Australia) for coding and analysis. Each transcript had a unique identifier comprising participant category, study site and sex to enhance anonymity and facilitate informed analysis. Outputs that could not be typed (photos from observations and participant diaries) were scanned and saved in a safe folder by the researchers, as they were reference materials during data analysis of the typed outputs.

### Data analysis

We used a framework analysis [41], informed by the Stakeholder Theory [31] and WHO governance framework [42,43] (Table 2). Framework analysis is adopted for research that has specific questions, a pre-designed sample and priory issues [41]^.^ The first step of framework analysis was listening to the recordings to familiarize the researchers with the information related to who the actors are in WASH in childcare centres, the networks that exist for the actors, the role of actors, the interests of the actors and where the actors get their authority. To ensure reliability, two researchers (an experienced qualitative researcher with WASH experience and an anthropologist) and 5 co-researchers, who collected the data participated in the development of a coding framework by reading the outputs imported in NVivo 12 software, participant diaries and photos independently to establish an inter-coder agreement. Once the initial coding framework was completed, the team met to discuss the themes generated and to reach an agreement on themes (Table 2). Two researchers proceeded with coding, charting, mapping and interpretation of transcripts.

**Table 2:** Themes for analysis.

Frameworks	Major theme	Sub-themes	Emerging themes
WHO framework.	Actors and networks.	• Categories of actors; • Networking of actors.	• Key & Non-key actors; • Possible, direct and desired networks; formal and informal networks.
Stakeholder theory.	Role of actors.	• Access to WASH; • Quality of WASH services.	• Access aspects- availability, affordability, acceptability, and adequacy and accessibility of WASH facilities and services; • Quality aspects- cleanliness, inclusion and safety WASH facilities and services.
	Authority for actors (Where do actors get authority).	• Elected; • Charisma; • Hybrid; • No authority.	
	Interests of actors.	• Child health; WASH provision.	

### Ethical considerations

The study was approved by AMREF Health Africa’s Ethics & Scientific Review Committee (ESRC), REF: AMREF-ESRC P747/2020. We also obtained approvals from National Commission for Science, Technology and Innovation (NACOSTI), REF: NACOSTI/P/20/7726. Approval was also obtained from the Liverpool School of Tropical Medicine (LSTM) and the African Population and Health Research Centre (APHRC) internal ethical review committee. All participants provided informed written consent before participating in an interview including consent for using photos and videos if there were any.

## 3. Results

We present findings of actors and networks, role of key actors, interest of key actors and authority of key actors in WASH service delivery in childcare centres.

Theme one: Actors and network of actors

### a) Actors

**Fig 4:**
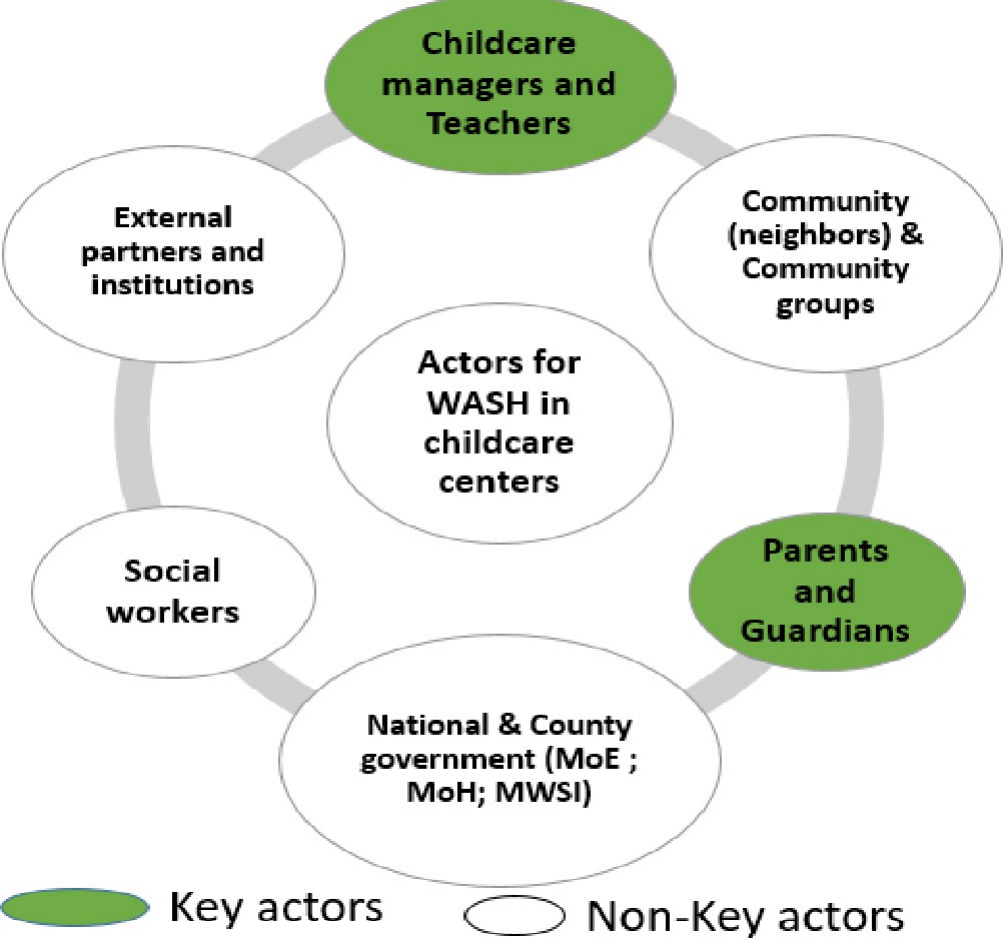
Results of actors in WASH service provision in childcare centres.

Social accountability actors included formal and informal actors with diverse networks. The actors’ authority was varied as interests varied from primarily profit-making to child’s health. Key actors in terms of access to WASH in childcare centres were parents and childcare managers/childcare centre owners as they were always close to the children in their daily activities and had a much bigger role in the provision of WASH services and related practices in childcare centres. Non-key actors included community members, community groups, neighbours, government ministries, social workers, institutions and external partners (Fig 4).

Childcare managers in both settlements, whose opinions reflected most childcare owners’ perceptions, described how they played a critical role in WASH service provision. Childcare owners always performed their roles in the provision of WASH services and in cases where an opportunity arose, they built the capacities of people around the centre on how to support the WASH activities at the centre. Centre owners also acknowledged the support from non-key actors like Community Based Organisations (CBOs), NGOs, and institutions like faith-based institutions and water vendors

*“It is my responsibility to ensure there is water*, *sanitation and hygiene products at the centre*. *I usually buy and sometimes we get support from CBOS*, *NGOs*, *churches*, *water vendors and even neighbours”* (IDI, Female Childcare manager, Korogocho).

Parents whose opinions reflected most parents’ perceptions also described themselves as key actors in water and sanitation in childcare centres, particularly in payment for WASH services. Despite the parents’ responsibility in WASH service delivery, they acknowledged that the government ought to be responsible, as the government sometimes worked together with external partners, institutions, community groups and water vendors in the WASH sector in the childcare centres.

*“Sometimes*, *I leave the child with the day-care owner and promise to return and give her money for services like sanitation*, *water…”* (IDI, Male Parent, Korogocho).

*“Government should be responsible for clean water… sometimes the government work with institutions*, *external partners and community groups or water vendors for access of water and sanitation at the centre”* (IDI, Female Parent, Viwandani).

### b) Networks of actors

**Fig 5:**
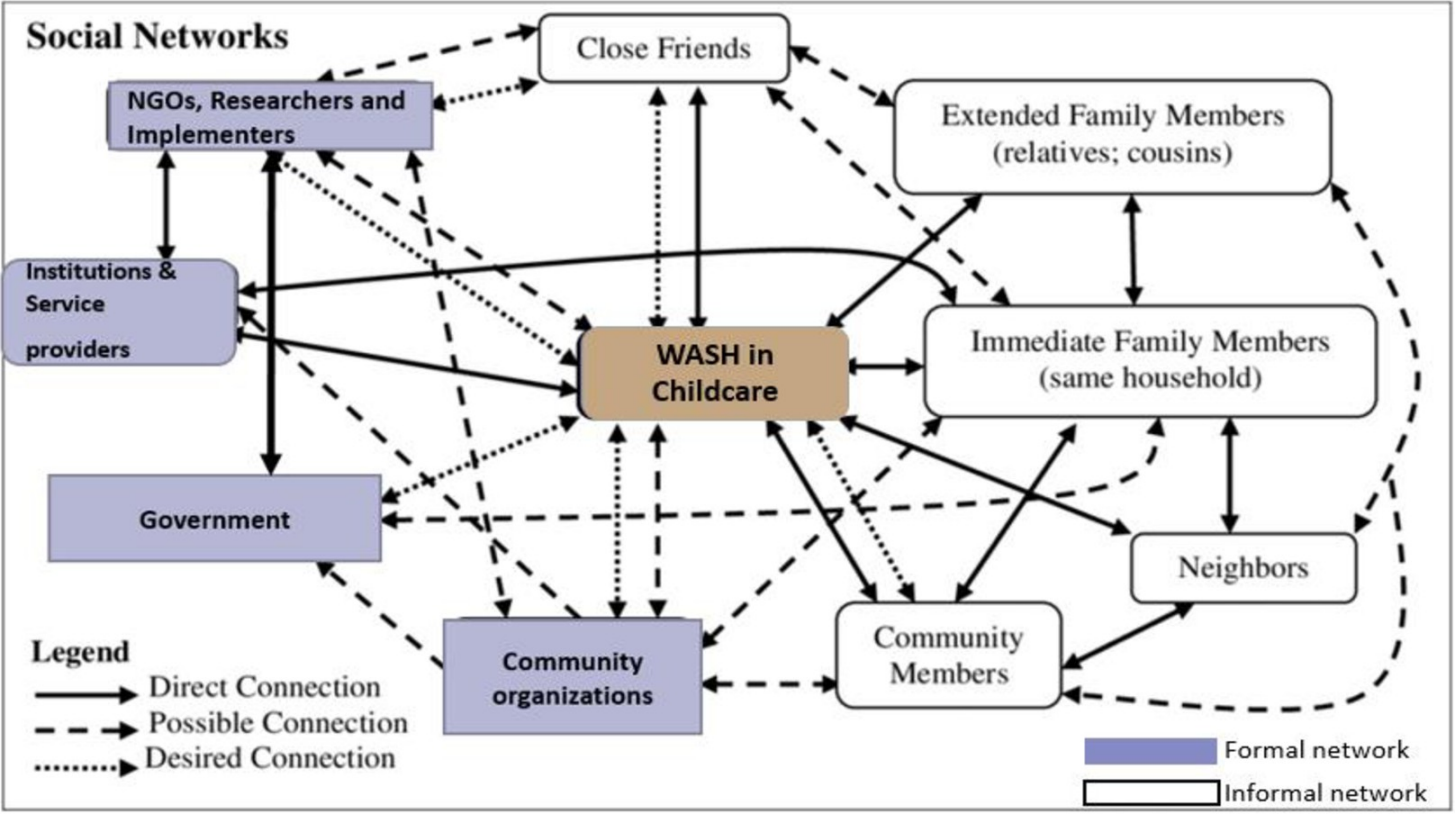
Results of network of actors in childcare centres.

Actors in childcare centres operated in direct, possible and desired networks. Actors who were providing WASH services in childcare centres directly without using agencies were described to have a direct network/connection. Actors who had the potential of providing WASH services because they were offering the services in the community were described to have possible connections/networks, while actors who were known by participants to be of great influence in WASH services but were not felt in the childcare centres were described to have desired networks. Some actors had multiple networks. The network of actors identified by the study participants is summarized in Fig 5.

There were formal and informal networks. Formal networks were embedded in formal rules and were few, unlike informal networks which were not embedded in formal rules. The networks had varying connections (Fig 5). Direct connections were portrayed through support in WASH service delivery from close friends, extended family members, immediate family members, neighbours, service providers and community members. Direct connections from actors meant that the actors were providing WASH services directly to the childcare centres.

*“We work with neighbours and friends; when I have no water or when I need sanitation facilities emptied*, *I sometimes get support directly from my close friend whom you met the other time or from my family members and relatives”* (IDI, Female Childcare manager, Korogocho).

*“When there are well-wishers around and I have no information on hygiene products being distributed by CBOs/NGOs/researchers or implementers… usually*, *community members will direct the partners to the centre"* (IDI, Female Childcare manager, Korogocho).

There were possible connections for WASH service provision from community organizations, non-governmental organizations (NGOs), researchers and implementers. The connections were deemed to be working in the communities but not directly with the childcare centres for access to WASH services. Therefore, there were possibilities for the actors to reach out directly to childcare centres as they were reaching out to communities at different scales.

*“Government is not directly helpful*, *as they work through community groups and community members who then avail water to the centre through water kiosks {water vendors}*. *CBOs*, *researchers*, *NGOs and other implementers are welcome in centres"* (IDI, Female Childcare manager, Viwandani).

There were desired connections for the WASH services provision in childcare centres from the government, community organizations, NGOs, researchers, implementers and community members. Key actors had desires for the actors with much impact in the community, who were referred to as desired networks.

“*We have a desire for the government to work with us directly in the provision of water and sanitation facilities*. (IDI, Female Childcare manager, Korogocho).

*“You know the government has much impact*, *yet*, *they are so rare around here*” (IDI, Male Parent, Viwandani).

*“If the government*, *community*, *NGOs*, *researchers and everyone work together to ensure there is WASH services in childcare centres*, *children will be healthy”* (IDI, Female Parent, Korogocho).

Efforts to interrogate actors and networks for individual WASH components did not show any difference in the networks and connections. We, therefore, did not duplicate figures of networks and actors for the separate aspects of water, sanitation and hygiene services and practices.

Theme two: Role of actors

Study participants described the role of key actors to be about access and quality of WASH services.

### a) Access

Access was described by availability, affordability, acceptability, distance and adequacy with respect to WASH facilities. Availability is the existence of WASH facilities and services; affordability is the cost-effectiveness of WASH facilities and services; acceptability is a tendency to agree with the standards, values and norms of WASH services; distance is the ability to reach the location of WASH services and adequacy is the ability to have enough WASH services [44,45].

#### Availability

Key actors (parents and childcare managers) described how their role revolved around the availability of WASH services in all circumstances, including having better water storage and holding meetings to identify better ways of availing WASH services in the childcare centres.

*“Right now*, *there is no water*, *yesterday water was scarce… But I ensure there is water for children at the centre by having good storage tanks"* (IDI, Female Childcare manager, Viwandani).

Parents described how the provision of WASH is also a government role. Unlike childcare managers, parents were selective in the centres where their children were admitted for their care. As such, they preferred admitting children to centres with facilities like WASH services.

*"It is the responsibility of the parent and the teacher to ensure there is WASH services in childcare centres… But they should work together with the government*.*"* (IDI, Male Parent, Korogocho).

*“I take my child to a childcare centre with good access to WASH facilities’* (IDI, Male Parent, Viwandani).

*"Parents can choose where to take their children*, *preferably centers with WASH facilities*. *For me*, *I have to take all children brought to me”* (IDI, Female Childcare manager, Korogocho).

#### Affordability

Centre managers provided water, sanitation and hygiene among other services, depending on how parents paid for the childcare services at the centre. There were many seasons where water was not affordable due to drought and pipe blockages, and many centre managers had devised strategies of ensuring water was always available through good storage and buying water from neighbouring villages, where they were sold at a cheaper price.

*“Water is not affordable when there is blockage of pipes… so what I usually do is to send some youth to buy cheaper in the nearby village*, *when I do not have enough in storage containers”* (IDI, Female Childcare manager, Viwandani).

*“When parents pay the weekly or sometimes daily fee*, *I buy and stock water in the evening since I am attending to children during the day”* (IDI, Female Childcare manager, Korogocho).

Many parents delayed payment of childcare service fees and sometimes did not pay in Korogocho, compared to Viwandani where many parents were receptive and could pay for the services needed. This is because many parents in Viwandani had a source of income from the casual jobs in the industrial area next to the settlement.

*“Many parents are receptive of payment for services because they go to work in those industries… when they earn their wages; they prioritize paying services for children… sometimes daily*, *weekly or monthly”* (IDI, Female Childcare manager, Viwandani).

*“Majority of parents do not have a source of income*, *they are just depending on wages from the dumping site and when they do not collect valuable goods*, *they are not able to pay for the services”* (IDI, Female Childcare manager, Korogocho).

#### Acceptability

The actors played a role in ensuring WASH services are acceptable. Parents rarely allowed their children to be in centres where the facilities were not acceptable. Parents who could not afford the payment of WASH services would end up attending childcare centres with unacceptable services due to a lack of options.

*“Parents like to know their children always have acceptable WASH facilities”* (IDI, Female Childcare manager, Korogocho).

*“Water*, *sanitation and hygiene services provided in some childcare centres are not acceptable but some parents take their children because they cannot afford centres with acceptable services”* (IDI, Female Parent, Korogocho).

#### Accessibility/Distance

Centre managers had innovative ways of ensuring a short distance to access WASH facilities. For instance, some had phone numbers for water vendors who would supply water to the centres. A centre owner whose sentiments represented many other managers described how childcare centres are located near basic facilities like water.

*"I have a phone number for the water vendors…*. *When I need water*, *I just contact them to deliver with a small fee"* (IDI, Female Childcare manager, Korogocho).

*“When I was looking for a room to start up the childcare centre*, *I was considerate to ensure it is located near water points in the community”* (IDI, Female Childcare manager, Viwandani).

#### Adequacy

There were consensus among key actors on the challenges related to the adequacy of WASH services. For example, there were seasons of water rationing in the community and inadequate sanitation facilities due to the economic status of parents, and as a result, children would share potties (portable toilets), even if it was not hygienic to share.

*"Most times*, *when I take the child*, *there is always water… I can recall the few times when the centre did not have water… The absence was due to actors who duplicated efforts*, *so there were more hand washing stations and no water for some days"* (IDI, Female Parent, Viwandani).

*“We advocate for adequate water and portable toilets…Though it is not always feasible because parents cannot afford for adequacy”* (IDI, Female Childcare manager, Korogocho).

### b) Quality

Study participants described how Actors in childcare centres valued quality WASH services, depicted by cleanliness, inclusion, and safety. Cleanliness is the tendency to have tidy WASH services; inclusion is the ability to have WASH facilities incorporating all children and safety is the tendency of WASH facilities to cause no harm to children in childcare centres [46,47].

#### Cleanliness

Actors were concerned with the cleanliness of WASH facilities for children at the centres.

*“We ensure that WASH facilities are clean*. *Sometimes one has no control over untidy sanitation facilities*, *when parents cannot afford to pay for services”* (IDI, Female Childcare manager, Korogocho).

#### Inclusion

Centre managers played a great role and offered equitable WASH services at the centres. However, it was not possible to have age and gender-appropriate WASH facilities due to the financial capabilities of parents, as such childcare owners could not always afford inclusive WASH facilities in their centres.

*“All children get to be included in access to water*, *sanitation and hygiene products*, *we do not discriminate”* (IDI, Female Childcare manager, Korogocho).

*"We know that it is important to have a sanitation facility separate for boys and girls but it is not possible since we cannot afford it"* (IDI, Female Childcare manager, Korogocho).

#### Safety

There were mixed reactions on the safety of WASH facilities. Safety of WASH facilities was of more concern to centre managers than to parents, as described by participants whose comments reflected the view of the majority of the study participants.

*"I am responsible for the safety of WASH in the daycare*. *I have to make sure that portable toilets are clean and safe for use by children”* (IDI, Female Childcare manager, Viwandani).

*“My concern is for children to access WASH facilities*, *I have not got much time to find out more about the safety"* (IDI, Male Parent, Korogocho).

Theme three: Authority of actors (Where actors get authority)

The authority for WASH service delivery was passion/charisma driven and sometimes it had a basis on values and norms/traditional approaches. For example, the passion and values of childcare providers towards childcare influenced childcare owners’ provision of WASH services.

*"I have the power to avail and teach children on WASH services*. *It is my passion and a talent as few people can clean public WASH facilities”* (IDI, Female Childcare manager, Korogocho).

*“My love for children and the tradition of ensuring children are served first*, *gives me authority and power to avail WASH services in the centres*, *even when I don’t have WASH services at home”* (IDI, Male Parent, Viwandani).

Parents described how other than centre managers, the government had power, yet they thought the power was more formal and rational rather than charismatic and hence not applicable in many childcare centres in many informal settlements.

*“Daycare owners have power on WASH facilities in the centres”* (IDI, Male Parent, Korogocho).

*"Government has the power to influence WASH service delivery*, *for example*, *they send community health volunteers to the centres to monitor the presence of WASH services in childcare centres*. *Also*, *right now {during the Covid-19 outbreak}*, *the government restricts children from going to school*, *that is why we are cautious when sending children to use centres’ sanitation facility”* (IDI, Female Parent, Korogocho).

Many centre managers guided children on WASH-related practices and kept their centres as clean as possible, despite their inability to control parents’ ability to pay for services. For example, in some instances, some parents could not pay for the collection of diapers, or soap/detergents and their power could be reduced in the provision of the service. However, centre managers contacted the parents and empowered them on the importance of WASH services for children, and encourage them to adopt a favourable payment mechanism (e.g. daily, weekly, monthly, or anytime when parents had money). In a few circumstances, childcare centres had banned parents who seemed to afford the services for their children but did not pay.

*"When parents fail to pay for services*, *I contact them on phone or look for them over the weekend and explain to them the importance of WASH services for children…*. *We met and discussed with them to adopt any payment mechanism including daily*, *weekly*, *monthly*, *or anytime they have money*. *This has helped in WASH payment and ultimately provision"* (IDI, Female Childcare manager, Viwandani).

*"There are some parents who by my assessment can afford WASH services but they neglect their role*. *For such parents*, *I do not allow their children to stay in the school if they stay long before parents pay for the services"* (IDI, Female Childcare manager, Korogocho).

Theme four: Interest of actors

The interest of actors, while providing WASH services in childcare centres was diverse ranging from income generation, access to WASH services by children, compliance with government regulations and promotion of child health and prevention of the spread of diseases. Unlike water and sanitation services, hygiene service was not deemed a source of income in both study sites. This assumption could be associated with advocacy and provision of hygiene services by many actors during the Covid-19 outbreak. Hygiene was also thought to be a measure of compliance with government regulations, unlike water and sanitation. The interest of actors is summarised in Table 3.

**Table 3:** Interests of WASH actors in childcare centres.

Theme	Viwandani	Korogocho
**Water** **Sanitation** **Hygiene**	**Parents’ perception**-Centre managers to earn money, Help children access to water, Centre managers to gain popularity. **Childcare managers’ perception**- Help children access to water, For the health and learning of children **Parents’ perception**- Centre managers to earn money, Centre managers to gain popularity, **Childcare managers’ perception**-Manage open defecation in the school and community, Help children access toilets easily, Control the Covid-19 rate of infection **Parents’ perception**- Prevent diseases like cholera and diarrhoea. **Childcare managers’ perception**—Ministry of Health regulations on Covid-19 preventive measures, Good health of children, Prevent contagious diseases like cholera and diarrhoea.	**Parents’ perception**—Centre managers to justify high fees for services at the centre, Centre managers to earn money, **Childcare managers’ perception—**Help children access to water, health of children **Parents’ perception**—Centre managers to earn money, Child care managers to justify a high cost of their services **Childcare managers’ perception** -Help children access toilets, For health of children **Parents’ perceptions**-Maintain cleanliness, Stop spread of diseases **Childcare managers’ perception—**Comply with Ministry of Health regulations on Covid-19 preventive measures, Promote good health for children.

There were diversities and dynamics in the interest of parents and childcare managers. The interests of parents and childcare managers were not mutually exclusive as actors had more than one interest. For example, a centre manager could have the interests of improving the health of children and compliance with the ministry of health regulations. Centre managers whose sentiments represented other managers described their interest in supporting children, ensuring the cleanliness of children and availing WASH services to children.

*“I like children and sometimes I use my own money to buy water for children when the parents have failed to provide WASH services… I have interest in ensuring cleanliness*, *complying with the ministry of health regulations and ensuring children are of good health”* (IDI, Female Childcare manager, Viwandani).

Childcare managers expressed the specific linkages between the health of children, WASH service provision and learning. They appreciated the fact that unhealthy children could not learn, and that WASH services and facilities affect the health of a child.

*“We promote and work for cleanliness so that children do not get dirty or even sick; sickly children cannot learn*.*”* (IDI, Female Childcare manager, Viwandani).

The interest of centre managers in both study sites was similar. However, the interests of parents slightly differed from the interest of centre managers. For example, parents’ interest included provision of water, sanitation and hygiene as a source of income, justification of a high cost of services and gaining popularity. However, the Center manager’s interests revolved around child health and helping children access WASH services.

## 4. Discussion

Access to WASH services in schools contributes to inclusion, dignity, and equity [13]. From a human rights perspective, WASH in schools is considered essential [2]. The Sustainable Development Goals (SDGs) implicitly highlights the need to expand WASH beyond household settings, in the effort to achieve universal and equitable access to safe and affordable drinking water, sanitation and hygiene for all [8]. This study builds on previous studies that have explored social accountability for WASH service delivery in schools [11,19]. Our study contributes to social accountability for WASH in childcare centres from the perspectives of both childcare centre managers and parents who are the key primary providers of WASH services, described in the short accountability route. A short accountability route enables users and service providers/ agents to develop networks that enable the achievement of a common goal [42]. Within the context of SDGs, the New Urban Agenda and AU’s Agenda 2063 as well as Kenya’s 2010 constitution and Vision 2030, all of which emphasize inclusive and equitable urbanization, it becomes important to understand existing social structures to build on in addressing the challenges of inclusivity and equitable service provision, especially for vulnerable and marginalised urban populations, including children.

For our study, parents and childcare providers developed networks to enable access and quality WASH service delivery in childcare centres. Little is known about social accountability for WASH service delivery in childcare centres in informal settlements, as such there was a need for governance diaries novelty approach. This approach enabled us to map social accountability structures for WASH in childcare centres, specifically understanding and documenting social actors and networks, their roles, authority and interests. Further, through stakeholder analysis, we analysed an array of stakeholders in WASH service delivery in childcare centres and identified and explored more of the key stakeholders/actors, who were parents and childcare managers. The key actors had a much bigger and primary role in the provision of WASH services in childcare centres. This is consistent with previous studies describing the importance of key actors and their roles in service delivery, the importance of understanding actors and linkages, identification of connections and networks in a community and assessing the authority within the networks on service delivery [48,49].

Actors in our study had direct, possible, or desired networks that were all-important for WASH service delivery, as they enhanced inclusion and reduced bureaucracies. As such, parents and childcare providers could operate in direct networks in promoting access to WASH services in childcare centres. This was reflected in previous studies that highlighted how networks provide flexible structures that are inclusive, informative, and outside the scope of direct bureaucratic control [50]. These flexible structures, which were informal, allowed the key actors, to leverage on existing desired networks and potential networks for access to WASH services by children in childcare centres. Within the context of other studies, this shows that the manifested absence of desired networks like government utilities in informal settlements, does not equal a latent lack of governance and social accountability [51], as resident actors (childcare owners and parents) did not fold their hands but rather stepped in to respond to the WASH needs of children through direct networks. However, parents and childcare owners acknowledged the responsibility of desired and potential networks in WASH service delivery, as these networks should focus on long-term coordination of organizations toward a common goal [52–54] and have the statutory obligations and state budgetary capacity to do so.

The role of actors ranged from promoting access to ensuring quality of WASH services delivery. For this study, some parents’ childcare managers lacked basic WASH resources and had limited space for making informed decisions on the rights to WASH services by children in childcare care centers. Despite the efforts by informal accountability structures, the lack of resources for sustained and robust long-term service provision has been offered as an explanations for better access than the quality of WASH service delivery [15,51] and consequently determine how users relate to services and how service providers respond to user’s preferences and priorities [12,13]. Once centre managers and parents become aware of the importance of WASH services, they are likely to be extraordinarily valuable social accountability agents in collaboratively making WASH services a daily experience.

Key actors had more power/authority over WASH service delivery for children in childcare centres, while the state had less power. From the key actors; service providers (childcare providers) and service users (parents), there were instances of weak social accountability because of indirect networks to the formal governance structures. The absence of formal actors in this important space is typified by parents and childcare providers not factoring formal WASH service provision into their primary and secondary focus but rather in their arrangements or seeking help from neighbours, community, community groups, community organizations, faith-based organizations or family. The challenge identified with these self-help service options is their amenability to exacerbate the disadvantage of the children it is intended to help, as services often reach users at a very high cost, with less effort on the equitable provision of WASH services to all. This finding relates to previous studies describing how in the absence of direct networks between government and key actors, decentralization of services means that local service providers may have more incentives to extract payments from service users like parents than to provide equitable services to children [53,54]. The social accountability landscape of WASH service delivery in childcare centres was filled with a broad array of actors with multiple connections and varying degrees of autonomy, sources of control and oversight. The interest of childcare managers who were providing WASH services varied from enhancing the health of children, and promoting cleanliness to income generation. This is consistent with previous studies, describing how the interest of actors varied, thus affecting WASH service delivery [17].

We conclude that WASH service provision in child care centres may be realised when key actors have a voice and work within networks to demand WASH services from desired networks including the government. We also conclude that developing more direct networks and converting desired and potential networks into direct networks in WASH service provision is critical for the success of WASH service delivery. Actors in WASH services in childcare centres may need to collaborate in identifying potential avenues for strengthening existing networks that enhance access and quality of WASH services in childcare centres. The study highlights the importance of strengthening networking among actors at all levels to facilitate smooth and effective delivery of WASH services to children in childcare centres. Lastly, we conclude that to achieve long-term sustainability of WASH service delivery, key actors may need to coordinate with one another and in relevant networks.

### Implications, future research and limitations

While the participants in this study showed awareness of issues around social accountability in aspects of actors, networks, roles, authority and interests, it is clear that further development and capacity building is needed to support centre providers and parents to establish desired networks with local government, NGOs, and other WASH implementing actors. This will raise the standard of WASH services in childcare centres. Furthermore, stakeholders working in childcare centres in the provision of WASH services should acknowledge the importance of existing actors and networks and capitalize on local arrangements with actors. Lastly, WASH actors ought to understand that there is no universally correct number of accountability linkages as it is situation-specific; depending upon the quality, not simply the number of connections, as such the quality of networks for centre managers and parents are always important.

Despite much progress made by studying actors and networks of organizations over the past years, there is still a considerable discrepancy between the acclamation and attention about the overall functioning of key actors and networks. Understanding the functioning of key actors and networks is important since only then can we better understand why actors and networks produce certain outcomes, irrespective of whether networks result from bottom-up or top-down processes. Embracing social accountability structures (actors and networks), authority and interest of actors allows for direct communication between users and service providers, as it recognizes residents as legitimate urban citizens and users of WASH services [21]. Additionally, understanding social accountability promotes constructive engagement among WASH actors on service provision, putting pressure on duty-bearers to justify their actions [55].

Future research on this topic could explore informal social accountability initiatives/models, which are widely acknowledged to have reduced power imbalances in multiple networks [20]. Additionally, future studies need to quantify the breadth of actors, networks, interests, authority and roles using network analysis. Social network analysis may be useful in characterizing and quantifying community differences related to community-based initiatives [56]. Finally, policymakers should build on such existing policies related to WASH service delivery in schools by ensuring that the policies are centred on components of WASH service delivery, whereas future WASH interventions should be framed in ways that are fundamentally empowering for parents and childcare centre providers.

Our study is not without limitations. This was a purely qualitative approach with a sample size of 24 study participants, as such results may not be generalized. The design was necessary for painting the depth of social accountability actors and networks for WASH in childcare centres within Nairobi’s informal settlements. A more holistic approach that combines qualitative and quantitative data, and integrates all stakeholders would be necessary for a broader understanding of the many aspects of the study, moving forward.

## Supporting information

S1 File (ZIP)

## Abbreviation

APHRC: African Population and Health Research Center
CAC: Community Advisory Committee
CBOs: Community Based Organisations
COREQ: Criteria for Reporting Qualitative research
ESRC: Ethics & Scientific Review Committee
FGDs: Focus group discussions
IDIs: In Depth Interviews
LSTM: Liverpool School of Tropical Medicine
NACOSTI: National Commission for Science, Technology and Innovation
NGOs: Non-Governmental Organisations
NUHDSS: Nairobi Urban Health and Demographic Surveillance System
SDG: Sustainable Development Goals
WASH: Water, sanitation and hygiene

## References

[1] Hughes R. C., Kitsao P.-, Bhopal S., Kimani E. W., Hill Z., and Kirkwood B. R., “Nairobi Early Childcare in Slums (NECS) Study Protocol: a mixed-methods exploration of paid early childcare in Mukuru slum, Nairobi,” pp. 1–7, 2020, doi: 10.1136/bmjpo-2020-000822 33344785 PMC7716665

[2] Wagner J. T., Wagner J. T., and Pramling I., “WASH from the START: Water, Sanitation and Hygiene Education in Preschool,” Int. J. Early Child., vol. 51, no. 1, pp. 5–21, 2019, doi: 10.1007/s13158-019-00236-5

[3] Black P. M. M. et al., “HHS Public Access,” vol. 389, no. 10064, pp. 77–90, 2018, doi: 10.1016/S0140-6736(16)31389-7.Advancing

[4] Ferguson A., Penney R., and Solo-Gabriele H., “A review of the field on children’s exposure to environmental contaminants: A risk assessment approach,” Int. J. Environ. Res. Public Health, vol. 14, no. 3, pp. 1–25, 2017, doi: 10.3390/ijerph14030265 28273865 PMC5369101

[5] Joint U., Programme M., and Water F. O. R., “DRINKING WATER, SANITATION AND HYGIENE IN SCHOOLS Global baseline report 2018 WHO / UNICEF JOINT MONITORING PROGRAMME FOR WATER SUPPLY, SANITATION AND HYGIENE,” 2018.

[6] Worrell C. M., Wiegand R. E., Davis S. M., and Odero K. O., “A Cross-Sectional Study of Water, Sanitation, and Hygiene-Related Risk Factors for Soil- Transmitted Helminth Infection in Urban School- and Preschool-Aged Children in Kibera, Nairobi,” no. March, 2016, doi: 10.1371/journal.pone.0150744 26950552 PMC4780697

[7] 2018 United Nations Children’s Fund (UNICEF) and World Health Organization, Drinking Water, Sanitation and Hygiene in Schools: Global baseline report 2018. 2018.

[8] WHO and UNICEF, “Core questions and indicators for monitoring WAUnited Nations Children’s Fund World Health OrganizationSH in health care facilities in the Sustainable Development Goals,” p. 28, 2018.

[9] UNICEF, “Baby WASH Programming: UNICEF Eastern and Southern Africa Learning Note 2020,” no. September, 2020.

[10] 2007 IRC, Towards Effective Programming for WASH in Schools. 2007.

[11] Wagner J. T. and Pramling Samuelsson I., “WASH from the START: Water, Sanitation and Hygiene Education in Preschool,” Int. J. Early Child., vol. 51, no. 1, pp. 5–21, 2019, doi: 10.1007/s13158-019-00236-5

[12] Friendly C. and Manual S., “Water, Sanitation and Hygiene (WASH) in Schools.”

[13] Mcmichael C., “Water, Sanitation and Hygiene (WASH) in Schools in Low-Income Countries: A Review of Evidence of Impact,” pp. 1–21, 2019, doi: 10.3390/ijerph16030359 30696023 PMC6388361

[14] Jiménez A., Livsey J., Åhlén I., Scharp C., and Takane M., “Global assessment of accountability in water and sanitation services using GLAAS data,” Water Altern., vol. 11, no. 2, pp. 238–259, 2018.

[15] Global WASH Cluster, “Modular Analytical Framework for Quality and Accountability,” p. 28, 2020.

[16] Satterthwaite D., “Editorial: A new urban agenda? Editorial: A new urban agenda?,” no. February, 2018, doi: 10.1177/0956247816637501

[17] Joshi S., “Community Participation & Ownership of Sanitation and Hygiene in Western Nepal,” Diacon. Univ. Appl. Sci., vol. 23, 2011.

[18] Accountability W., “Wash Accountability,” 2020, [Online]. Available: www.unicef.org.

[19] Antwi-agyei P. et al., “Water, sanitation and hygiene (WASH) in schools: results from a process evaluation of the National Sanitation Campaign in Tanzania,” pp. 140–150, 2017, doi: 10.2166/washdev.2017.159

[20] Chumo I., Kabaria C., Muindi K., Elsey H., Phillips-howard P. A., and Mberu B., “Informal social accountability mechanisms for water sanitation and hygiene (WASH) in childcare centres in Nairobi City County ‘ s informal settlements,” Urban Gov., no. July, 2022, doi: 10.1016/j.ugj.2022.07.001

[21] Velleman Y., “Social Accountability: Tools and Mechanisms for Improved Urban Water Services,” Discuss. Pap., pp. 1–40, 2010.

[22] Chikozho C., Kadengye D. T., Population A., Wamukoya M., Population A., and Health N. U., “Leaving no one behind? Analysis of trends in access to water and sanitation services in the slum areas of Nairobi, 2003–2015,” no. June, 2019, doi: 10.2166/washdev.2019.174

[23] Mafuta E. M. et al., “Participatory approach to design social accountability interventions to improve maternal health services: a case study from the Democratic Republic of the Congo,” Glob. Heal. Res. Policy, vol. 2, no. 1, pp. 1–16, 2017, doi: 10.1186/s41256-017-0024-0 29202072 PMC5683322

[24] Schaaf M. and Freedman L. P., “Unmasking the open secret of posting and transfer practices in the health sector,” Health Policy Plan., vol. 30, no. 1, pp. 121–130, 2015, doi: 10.1093/heapol/czt091 24324005 PMC4287189

[25] Hamal M., De Cock Buning T., De Brouwere V., Bardají A., and Dieleman M., “How does social accountability contribute to better maternal health outcomes? A qualitative study on perceived changes with government and civil society actors in Gujarat, India,” BMC Health Serv. Res., vol. 18, no. 1, pp. 1–15, 2018, doi: 10.1186/s12913-018-3453-7 30134881 PMC6106761

[26] Eid Y. Y., Khalifa M. A., and Azouz N., “Good Urban Governance of Informal Settlements in Metropolitan Areas: Case Study of the Informal Settlement of Ezzbet Al- Haggana, Cairo-Egypt,” World Sustain. Build. Conf., p. 11:20, 2014, [Online]. Available: https://www.researchgate.net/publication/269230666_Good_Urban_Governance_of_Informal_Settlements_in_Metropolitan_Areas_Case_Study_of_the_Informal_Settlement_of_Ezzbet_Al-_Haggana_Cairo-Egypt.

[27] Diachok M. and of Sanjay Agarwal M. D., Rasmus Heltberg, “Scaling-up Social Accountability in World Bank Operations,” Soc. Dev. Dep. World Bank, pp. 1–12, 2009.

[28] Camargo C. B. and Stahl F., “Social accountability A practitioner’s handbook,” p. 65, 2016.

[29] Khotami M., “The Concept Of Accountability In Good Governance,” vol. 163, no. Icodag, pp. 30–33, 2017, doi: 10.2991/icodag-17.2017.6

[30] R. K. Mitchell and D. J. Wood, “Toward a Theory of Stakeholder Identification and Salience: Defining the Principle of Who and What Really Counts Author (s): Ronald K. Mitchell, Bradley R. Agle and Donna J. Wood Published by: Academy of Management Stable URL: https://www.jstor.o,” vol. 22, no. 4, pp. 853–886, 1997.

[31] Morad N., “Stakeholder theory: origins, developments and contributions to the field of business and society Théorie des parties prenantes: origines, développements et contributions au champ de l ‘ entreprise et la société,” pp. 1–18, 2021.

[32] Dawe N. K. and Ryan K. L., “The Faulty Three-Legged-Stool Model of Sustainable Development,” Conserv. Biol., vol. 17, no. 5, pp. 1458–1460, 2003, doi: 10.1046/j.1523-1739.2003.02471.x

[33] Tong A., Sainsbury P., and Craig J., “Consolidated criteria for reporting qualitative research (COREQ): A 32-item checklist for interviews and focus groups,” Int. J. Qual. Heal. Care, vol. 19, no. 6, pp. 349–357, 2007, doi: 10.1093/intqhc/mzm042 17872937

[34] Munyewende P. O., Rispel L. C., and Rispel L. C., “Using diaries to explore the work experiences of primary health care nursing managers in two South African provinces,” vol. 9716, 2015, doi: 10.3402/gha.v7.25323 25537937 PMC4275646

[35] Bishop S., “Using Water Diaries to Conceptualize Water Use In Lusaka, Zambia.”

[36] Tkacz N., “Data diaries: A situated approach to the study of data,” 2021, doi: 10.1177/2053951721996036

[37] A. Lanerolle, Indra de; Walton, Marion; Schoon, “Izolo: mobile diaries of the less connected,” Mak. All Voices Count Res. Report, no. November, p. 31, 2017, [Online]. Available: https://opendocs.ids.ac.uk/opendocs/bitstream/handle/20.500.12413/12837/MAVC_CIPGv2_Final_online.pdf.

[38] Beguy D. et al., “HDSS Profile HDSS Profile: The Nairobi Urban Health and Demographic Surveillance System (NUHDSS),” pp. 1–10, 2015, doi: 10.1093/ije/dyu251 25596586

[39] Emina J. et al., “Monitoring of Health and Demographic Outcomes in Poor Urban Settlements: Evidence from the Nairobi Urban Health and Demographic Surveillance System,” vol. 88, pp. 200–218, 2011, doi: 10.1007/s11524-011-9594-1 21713553 PMC3132229

[40] “The Nairobi City County Childcare Facilities Act, 2017.”.

[41] S. R. and S. R. Nicola K Gale1*, Gemma Heath2, Elaine Cameron3, “Using the framework method for the analysis of qualitative data in multi-disciplinary health research,” pp. 1–8, 2013. 10.1186/1471-2288-13-117 PMC3848812 24047204

[42] Fox J. A., “Social Accountability: What Does the Evidence Really Say?,” World Dev., vol. 72, no. December, pp. 346–361, 2015, doi: 10.1016/j.worlddev.2015.03.011

[43] Fox J. and Fox J., “GPSA WORKING PAPER SERIES SOCIAL ACCOUNTABILITY: WHAT DOES THE EVIDENCE REALLY SOCIAL ACCOUNTABILITY: EVIDENCE REALLY SAY?,” no. 1, pp. 1–58, 2014.

[44] Thiede M., Akweongo P., and McIntyre D., “Exploring the dimensions of access,” Econ. Heal. Equity, pp. 103–123, 2007, doi: 10.1017/CBO9780511544460.007

[45] Scheffler E., Visagie S., and Schneider M., “The impact of health service variables on healthcare access in a low resourced urban setting in the Western Cape, South Africa,” African J. Prim. Heal. Care Fam. Med., vol. 7, no. 1, 2015, doi: 10.4102/phcfm.v7i1.820 26245611 PMC4656938

[46] WHO, “Introducing the WHO Quality Toolkit. Supplemental Overview,” 2022, [Online]. Available: https://apps.who.int/iris/bitstream/handle/10665/353566/9789240043879-eng.pdf?sequence=1&isAllowed=y.

[47] Ministry of Education K., “REPUBLIC OF KENYA EARLY CHILDHOOD DEVELOPMENT SERVICE STANDARD GUIDELINES FOR KENYA,” 2006.

[48] Accountability R., “New Roles for Accountability Actors,” pp. 77–78, 2005.

[49] “Understanding Factors and Actors to Achieve Sustainable Drinking Water Systems in Kitui County, Kenya,” 2018.

[50] Isett K. R., Mergel I. A., Leroux K., Mischen P. A., and Rethemeyer R. K., “Networks in Public Administration Scholarship: Understanding Where We Are and Where We Need to Go,” pp. 157–173, doi: 10.1093/jopart/muq061

[51] Leroux K. et al., “Informal Accountability in Children ‘ s Service Networks: The Role of Frontline Workers Informal Accountability in Children ‘ s Service Networks: The Role of Frontline Workers,” Hum. Serv. Organ. Manag. Leadersh. Gov., vol. 43, no. 3, pp. 188–204, 2019, doi: 10.1080/23303131.2019.1637804

[52] Klijn E.-H. and Koppenjan J., “Complexity in Governance Network Theory,” Complexity, Gov. Networks, vol. 1, no. 1, p. 61, 2014, doi: 10.7564/14-cgn8

[53] Harm F., “Accountability in Local Service Delivery The Tuungane Community Scorecard Approach Policy and Practice Briefing Paper Prepared by Guillaume Labrecque and Isatou Batonon Accountability in Local Service Delivery—The Tuungane Community Scorecard Approach.”

[54] UNDP, “Social Accountability in a Changing Region—Actors and Mechanism,” pp. 11–24, 2010, [Online]. Available: https://www.shareweb.ch/site/DDLGN/Documents/Social_accountability_changing_region_Report_English_Gov_Week_Cairo_March_14.pdf.

[55] Schaaf M., Topp S. M., and Ngulube M., “From favours to entitlements: Community voice and action and health service quality in Zambia,” Health Policy Plan., vol. 32, no. 6, pp. 847–859, 2017, doi: 10.1093/heapol/czx024 28369410 PMC5448457

[56] Feinberg M. E., Riggs N. R., and Greenberg M. T., “Social Networks and Community Prevention Coalitions,” vol. 26, no. 4, 2005, doi: 10.1007/s10935-005-5390-4 15995800

