# Supplementary material for: Mapping social accountability actors and networks and their roles in water, sanitation and hygiene (WASH) in childcare centres within Nairobi’s informal settlements: A governance diaries approach
Source: PLoS One. 2022 Nov 15;17(11):e0275491. doi: 10.1371/journal.pone.0275491 (PMC9665391; doi:10.1371/journal.pone.0275491)
Supplement: S1 File — (ZIP) [file pone.0275491.s001.zip › Anonymized Transcripts Plos (SAM)_Oct 2022/IDI 1_Stydy Site A (Female Respondent).docx]

**PARTICIPANT: Parent of a child attending childcare center**

**AREA: Study site A**

**Key**

**M: Moderator**

R: Respondent

**M: Welcome to today’s interview. We are doing an IDI on Arise project. The respondent is a parent of a child under five years. I’m your moderator, FS1 and the note-taker is FS2. Welcome. Welcome to the discussion. So maybe you tell me about your experience with regards to water, sanitation and hygiene at the daycare your child goes to. Maybe we begin with water, what is your experience with the water at the daycare?**

R: It is good.

**M: So what is your experience with water?**

R: Water is okay. It is available.

**M: So they get water at the daycare?**

R: Yes.

**M: Okay, is it in the tanks or just tap water?**

R: It is in the tanks and they also have a tap.

**M: Okay, and do they have water shortages or is it always available at all times?**

R: It is always available because they have a borehole.

**M: And what can you tell me about the toilet at the daycare?**

R: The toilets are also okay.

**M: Are they enough for the children or are they few?**

R: They are enough.

**M: Okay, how many toilets are they?**

R: I know there are two outside.

**M: Do they belong to the school?**

R: Yes, they belong to the school but outside.

**M: so they have two toilets?**

R: Yes, they also have some up there.

**M: And what can you say about the hygiene of the toilets?**

R: They are okay.

**M: Okay, and do the children wash their hands at the daycare?**

R: Yes, they do.

**M: And what do they use to wash their hands?**

R: They use soap.

**M: They use soap?**

R: Mhm

**M: That’s good. Who is accountable to ensure that there is water in the daycare centers?**

R: The school employees.

**M: Which employees – are there employees…**

R: The ones who cook for the children.

**M: So it’s their responsibility to ensure that the children have access to drinking water?**

R: And even the head of the school.

**M: So what do the employees and the head of the school do to ensure that the children access water?**

R: Accessing water?

**M: Yes, what do they do to ensure that these children access water while at the daycare?**

R: They put the water out there for the children to use. They do have water in a bucket.

**M: Is that for hand washing or drinking?**

R: They also have drinking water. We have drinking water and some for hand washing.

**M: And who ensures that the children have access to water for hand washing?**

R: The head of the school.

**M: Is there anyone else?**

R: Also the employees.

**M: And sanitation; whose responsibility is it to ensure that the children have access to the toilets while at the daycare?**

R: The head of the school.

**M: Okay, and what does the head of the school do to ensure that the children have access to the toilet?**

R: They construct the toilet.

**M: Okay, is there anyone else accountable to ensure that the children have access to water, toilet and hand washing?**

R: The parents as well.

**M: And what is the role of the parents in ensuring that the children have access to drinking water, hand washing, soap and toilet services?**

R: You go there are ask the children to wash their hands. You also place the drinking water – do you mean at the daycare?

**M: Yes, at the daycare.**

R: If there is no water at the daycare the parents will just say it and the head of the school will provide them.

**M: That’s good. So when I say WASH I mean three things, water, sanitation and hygiene. So how do you know that your child has access to WASH services while at the daycare center? You said that your child is in pp1, right?**

R: Mhm

**M: So how do you know that the child has access to drinking water, hand washing and sanitation services?**

R: I do ask her when she comes back in the evening.

**M: And apart from asking her, how else do you know that the child has access to these services?**

R: You can go to the daycare and see for yourself.

**M: Okay, is there any other way?**

R: No.

**M: And how do you know that the child has access to drinking water at the daycare?**

R: It is not easy to know that because it is always available.

**M: Okay, have you ever done anything to know whether the water is safe for the child to drink or to ensure that it is of high quality?**

R: I haven’t.

**M: And how do you know that your child has access to good hand washing while at the daycare or that they get quality hand washing services at the daycare?**

R: I do ask her.

**M: And how do you know that she has access to quality sanitation services?**

R: I just ask her.

**M: And how does the child report on WASH at the daycare? Maybe the child is at the daycare and wants to report on toilet, hand washing or water, how can they give this report?**

R: They can tell the teacher.

**M: Okay, any other way?**

R: They can tell the parents to report to the teachers.

**M: Okay, and how can the child report that to you as a parent or what do they always do to provide the report?**

R: They just come and explain to you. Sometimes you ask her.

**M: Okay, that’s good. And how does the school administration view these reports on WASH from the children? Let’s say the child provides – you said that the child can report at the daycare about water or sanitation, so how will the school administration view the report?**

R: When they are told they will take action.

**M: Let’s say that she has said that the drinking water isn’t safe, what would the school do or how will they take the report?**

R: They will take it in a negative way.

**M: Negatively?**

R: Mhm

**M: Why?**

R: They will ask why the child is saying the water is dirty.

**M: Okay so they won’t take the report positively. And let’s say they report that they don’t know how to use the toilet, how will the school administration view the report?**

R: They will just take action.

**M: What can they say if the child says that they have difficulty in using the toilets available?**

R: They will guide her.

**M: They will guide her on how to use them?**

R: Yes.

**M: Okay, and let’s say she reports that she cannot access soap for hand washing, how will the school administration view that report?**

R: They can provide them.

**M: The soap?**

R: Mhm

**M: And is there any other way the school can view the report from the child while at the daycare?**

R: On?

**M: Maybe is there another way the school administration will view the report by the child apart from the ones you’ve mentioned?**

R: Taking action…

**M: Or how can they view the reports from the child apart from what you’ve told me?**

R: None.

**M: Okay, and in case a child has a difficulty in using WASH facilities, what would you do or what can you advise them to do? If a child reports to you that they don’t have drinking water or soap for hand washing at the daycare, what would you do?**

R: You go and talk to the teachers.

**M: Okay, is there anything else you can do?**

R: Or you can give her soap to take to school.

**M: Okay, and what have you been doing whenever the child gives you such reports?**

R: You just go and tell them.

**M: And has she ever given you such a report?**

R: No.

**M: Okay, now let’s say the child has difficulty in using WASH services, who do you think is expected to solve that problem while at the daycare?**

R: The employees of the school.

**M: What should they do?**

R: They should ensure that the toilet is clean and the drinking water is safe.

**M: Is there anyone else apart from the employees?**

R: The head of the school.

**M: Okay, is there anyone else?**

R: No.

**M: Okay, and you’ve told me that it’s the employees of the school and the head of the school; why do you think they are the ones responsible?**

R: Because the head of the school owns the school and the employees are employed to take care of those services.

**M: And when the child is at home who is responsible for those services?**

R: The parent.

**M: Why the parent?**

R: Because the parent will be the closest to the child.

**M: And why do you think the teacher of the child also expects you to be responsible for WASH of the child?**

R: The parent is responsible because she is the parent.

**M: Is there any other reason why the teacher expects you to be accountable for WASH services of the child?**

R: No.

**M: We’re moving on well. I would like to know, what do you think the teacher expects your role to be with regards to WASH?**

R: When the hands of the child are dirty you can tell them to wash their hands.

**M: Okay, and what about sanitation?**

R: You can show her the toilet.

**M: What do you show her?**

R: You show her where the toilet is.

**M: And what about water?**

R: You ensure that the water is clean.

**M: Is there any other role the teacher expects you to play?**

R: No.

**M: Okay and the roles you play like on hand washing and showing the child the toilet, how are you accountable in your roles to ensure that the toilet is clean and they wash their hands using soap?**

R: You ensure that the water to fetch is clean.

**M: Okay, and what about the toilet?**

R: You also clean the toilet.

**M: Okay, and what about hand washing?**

R: You just provide her with soap and water to wash her hands.

**M: Okay, do you think there is something you can do to achieve your roles better?**

R: [laughs]

**M: You’ve said that you do clean the toilet. So what do you think can help you clean the toilet better than you do currently?**

R: I just use the toilet detergent.

**M: And you also said that you ensure that water is clean, so what can be done to make your work easier and access clean water easily?**

R: Maybe you just take – when you figure that the water is not very clean then you boil it.

**M: That’s good. And who is responsible to enforce or ensure discipline on WASH rules on the parents and teachers?**

R: And the teachers?

**M: Yes, who is accountable to ensure that there is discipline or enforce the rules for the teachers and parents with regards to WASH?**

R: Ourselves.

**M: Yourselves?**

R: Mhm

**M: Maybe if we start with the teachers, who should ensure that the teachers enforce the WASH policies at the daycare?**

R: The parents can tell them.

**M: Who else can tell the teachers?**

R: The heads of the school.

**M: Okay, is there anyone else?**

R: No.

**M: So how should the heads of the daycare enforce the rules for the teachers to achieve their roles better?**

R: They just explain to them.

**M: Okay, and you’ve also told me that as parents you can enforce the rules on the teachers; so what do you do to ensure that the teachers ensure that the children have access to sanitation services?**

R: That’s tough.

**M: It’s tough?**

R: Mhm

**M: You also told me that as parents you can also talk to the teachers, right?**

R: Mhm

**M: So who can ensure that as parents when the children are at home they have access to toilet and clean water?**

R: No one.

**M: So you don’t have anyone among the parents?**

R: Mhm

**M: I want to ask you about policymakers – do you know who policymakers are?**

R: No.

**M: So policymakers are for example the people who tell the schools that they have to have toilets or drinking water for the children. So how are the policymakers accountable to ensure that the WASH services are available at the daycare centers?**

R: They do check.

**M: What do they check?**

R: They check whether water is available. They even ask them where they get the water from.

**M: And then? After they ask how do they follow up? Let’s say they check and find that there is no water, what would they do?**

R: They can tell them how they can access water.

**M: Okay, and what do they do in the toilets?**

R: They check whether the toilet is clean.

**M: And if they find that it’s not clean?**

R: They tell them to clean it.

**M: Okay, and what do they do about hand washing?**

R: They ensure that there are hand washing facilities available.

**M: And now the teachers and parents both participate to ensure that the children have access to toilet or drinking water, right?**

R: Mhm

**M: Should they be involved in ensuring that the children have access to water, sanitation and hand washing services?**

R: Yes.

**M: Okay, to what degree should they be involved?**

R: They just go.

**M: And in your opinion do you think that the parents and teachers should be highly involved, moderately involved or involved in a small way?**

R: They should be highly involved.

**M: Why do you say so?**

R: They should be highly involved because the children belong to us.

**M: Is there any other reason?**

R: No.

**M: And as a parent, if you compare how you are involved in the WASH of your child and how the other parents are involved with their child, how can you compare that?**

R: It is just okay.

**M: So I would like to know whether you are more involved than the other parents or are the other parents more involved or are you involved at the same rate.**

R: At the same rate.

**M: And how the teachers in your child’s daycare are involved on WASH services and the other teacher’s involvement, can you say that the teachers of your child’s daycare are more involved with WASH than the other teachers or not?**

R: They just get involved the same way.

**M: That’s good. And what is the voice of the parents to the policymakers and the service providers?**

R: Parents…

**M: Maybe what do the parents say with regards to WASH to the teachers? WASH refers to water, sanitation and hygiene; so what do the parents say to the teachers with regards to water, sanitation and hygiene?**

R: They just say that water should be available.

**M: Water should be available?**

R: Mhm

**M: What else?**

R: That’s all.

**M: And do you think that the say is required or needed?**

R: It is needed.

**M: How can it help?**

R: [silence]

**M: Okay, and what do the parents say to the policymakers?**

R: They just tell them to ensure that the services are available.

**M: And let’s say you had an option of taking your child to a different daycare, would you take her there?**

R: No.

**M: Why?**

R: She will just go to the same daycare.

**M: Why would you want her to remain in the same daycare?**

R: Because the hygiene at that daycare is well maintained.

**M: Okay, is there any other reason?**

R: No.

**M: Okay, I would now like us to talk about the accountability models. So I would like to know how the users, service providers and policymakers ensure the quality of WASH in the daycare center.**

R: In the community?

**M: In the daycare centers.**

R: Mhm

**M: What do they do?**

R: They just ensure that water is available.

**M: Okay, I would like to know how the parents, teachers and policymakers ensure that the children at the daycare have access to toilet, drinking water and hand washing.**

R: They consult one another.

**M: They consult?**

R: Mhm

**M: About?**

R: How to get the services like constructing toilets and getting water.

**M: Is there anything else they do?**

R: No.

**M: And what would you recommend to the parents, teachers and policymakers for the WASH services to be improved or be easily available? Okay, what can you tell the parents, teachers and policymakers to help improve the WASH services at the daycare? In your opinion what do you think can be done to improve sanitation services at the daycare?**

R: If water can be available it can be good.

**M: So what should be available?**

R: Mhm

**M: And what about the toilet; what can you say?**

R: As long as the toilet is clean.

**M: So the toilet should be cleaner than it is?**

R: Yes.

**M: Okay, and how can that be done?**

R: They just remind the children to go to the daycare with water.

**M: Okay, and what can be done for the water to be available at all times?**

R: They can have water storage tanks.

**M: Okay, and on hand washing, what can be done to improve on hand washing?**

R: They should have soap.

**M: Okay, and do you have accountability model that you would recommend to help improve WASH services?**

R: Water and soap being available.

**M: Why do you recommend that water and soap be available?**

R: Without water they cannot clean themselves or if there is not soap they won’t wash their hands.

**M: Okay, how will the model affect the health of the children at the daycare center?**

R: Their health will improve.

**M: It will improve?**

R: Yes.

**M: Okay, is there any other way it will affect their health?**

R: No.

**M: Okay, we’re almost coming to the end. So maybe you tell me about the rules with regards to WASH services in the daycare centers.**

R: The rules in the daycares?

**M: Yes, the rules in the daycares with regards to WASH. What rules does the daycare have with regards to sanitation?**

R: [silence]

**M: Maybe there are rules they have in place for the children when they use the toilet or after using the toilet or even on hand washing. Are there any rules?**

R: After they use the toilet they flush it properly.

**M: The children?**

R: Yes, when they use the toilet.

**M: so they have a rule that after using the toilet the children flush the toilet?**

R: Mhm

**M: So the children are the ones who flush the toilet after using it?**

R: Yes.

**M: Okay, is there any other rule on sanitation at the daycare?**

R: No.

**M: What about on hand washing; what rules do they have?**

R: Before you enter, you have to wash your hands.

**M: Where?**

R: After using the toilet you wash your hands.

**M: And what about a rule on drinking water at the daycare?**

R: They just have water available so I don’t know of the rules.

**M: And I would like to know the policymakers, what do they do to ensure that the children at the daycare have access to sanitation, water and hygiene services. How are they accountable to ensure that the children have access to these WASH services?**

R: They check if they are finished and add some.

**M: What?**

R: Water.

**M: Drinking water or water for hand washing?**

R: Drinking and hand washing.

**M: What about toilet?**

R: They also ensure that there is water by the toilet so that they wash their hands after using the toilet.

**M: And is there strategy they have in place to ensure that you also participate in the – how do the policymakers ensure that as teachers you also participate in the WASH service provision at the daycare?**

R: When they come to the school and ask the teachers or they can see the services are available.

**M: Okay, and can the teachers report to the policymakers for example if there is something they want to tell them; is there a channel the teachers can use to talk to them?**

R: They can talk to them.

**M: Using what channel?**

R: You just tell them if there is a challenge.

**M: And let’s say if there is a concern, do they have a channel for reporting on concerns?**

R: Yes, they can give out their phone numbers.

**M: That’s good. I would now like us to talk about the daycare centers, daycare owners and service providers like Nairobi water. So I will read you statements and then you will tell me how these daycare owners ensure that the services are available in the daycare centers. So what do the teachers do to ensure that they are trusted with the children’s access to the toilet, water and sanitation?**

R: They place them out there.

**M: So what do they place out there?**

R: Water. When the children go to the daycare they ask them to wash their hands before getting in.

**M: So when you see that you trust them?**

R: Yes.

**M: Is there anything else they do for you to trust them?**

R: They also have drinking water available.

**M: And what do the teachers do to ensure that they can advise you as parents with regards to the use of WASH services?**

R: Advising us at the daycare?

**M: As in what do they do to ensure or what channel do they use to advise you as parents on sanitation, water and hygiene?**

R: They guide the children on how to use the toilets.

**M: So they guide the children?**

R: Yes.

**M: And what do the teachers do to ensure that there is transparency in how they provide these WASH services to the children?**

R: They just place them there.

**M: Where?**

R: Just in the buckets out there.

**M: And what do they do to ensure that there is transparency in sanitation at the school?**

R: They ensure that there are enough toilets.

**M: And what do the teachers do to ensure that there is equity maybe in the use of these WASH services?**

R: Accessing?

**M: What do they do to ensure that there is equity when the children need water – maybe a child has been discriminated on accessing water; so what do they do to ensure that there is equity in access to water among the children?**

R: They just show them and tell them that whoever wants water can get it.

**M: What do they show them?**

R: Water.

**M: And what do they do to ensure that there is responsiveness in access to these WASH services?**

R: They just show the children.

**M: And what do the teachers do to ensure that the WASH services are affordable?**

R: I don’t know about that.

**M: I would like to know what they do to ensure that for example sanitation services are affordable to you or the water or soap are also affordable to you.**

R: The toilet is available at the daycare.

**M: Okay, and what about water and soap?**

R: They do have liquid soap.

**M: And do you pay for that?**

R: No.

**M: Okay, and are you also required to supply things like tissues?**

R: Yes.

**M: Okay, and what do you do to ensure that maybe the tissue is affordable?**

R: We do buy them.

**M: And what do they do to ensure that the water is of high quality and the sanitation services are of high quality?**

R: They treat the water.

**M: And what do they do about the toilets?**

R: They just clean it.

**M: And what do they do about hand washing?**

R: They ensure that soap is available.

**M: And what do they do to ensure that these WASH services are easily available?**

R: They are always available.

**M: We are on to the final part where we are going to talk about parents. So I will read statements to you and you will tell me how as parents you ensure that they are available. So as a parent how do you adhere to the payment of the WASH services?**

R: We don’t pay for them. I don’t know whether we pay through the school fees or anything.

**M: So you don’t pay for sanitation, water and hygiene services?**

R: No, we don’t pay for them.

**M: And what do you do as parents to follow up on the quality of the drinking water, toilet and hand washing?**

R: When the children are at the daycare?

**M: Yes.**

R: We just ensure – you tell the child to wash her hands after using the toilet.

**M: Okay, and what do you do to ensure that the water that the child drinks is of good quality?**

R: We ensure that the water is treated.

**M: So you do ensure that you have treated water? I would like to know how you follow up on the cleanness of the toilet at the school; as a parent how can you ensure that?**

R: I ask the child.

**M: So that’s on toilets?**

R: Mhm

**M: And what do you do about water to ensure the quality of the water the children drink?**

R: You also ask the child if they drink clean water.

**M: And how do you ensure the quality of hand washing?**

R: You just ask the child if they do wash their hands.

**M: And what do you do to get involved in the management of the WASH services at the daycare?**

R: [silence]

**M: What do you do to ensure that as a parent you know what is going on with regards to sanitation and maybe you consult with the parents and such like things?**

R: You just go and if you don’t find any you ask the teacher.

**M: That’s good. And what do you do to ensure that there is accountability in the use of WASH services?**

R: You can just go and check.

**M: Okay, so what would you do if you find that the services are bad?**

R: You tell the teachers.

**M: And what will the teachers do?**

R: They will solve it.

**M: And if they don’t correct the mistakes what would you do?**

R: That’s tough.

**M: And what do you do to ensure that you can raise your concerns with regards to toilet, water and hygiene as parents?**

R: If you don’t find the services you just tell the teachers.

**M: Is there any other way?**

R: No.

**M: Thank you. Now onto our final question, how did corona affect the WASH service provision at the daycare? Let’s start with water, how did corona affect water service at the daycare?**

R: It didn’t affect.

**M: So the same way they were accessing water before corona and right now are all the same?**

R: Yes.

**M: And how did corona affect sanitation services?**

R: It’s just okay.

**M: If you compare before corona and right now are they the same?**

R: Yes.

**M: And how did corona affect hand washing at the daycare centers?**

R: Before corona hand washing wasn’t done frequently but corona has made it be done frequently.

**M: And if you look at the services we have talked about of sanitation, water and hygiene at the daycare centers, which one should be given a priority?**

R: Hand washing.

**M: Why?**

R: Because sometimes the child touches something.

**M: What happens if the child touches something?**

R: If someone had corona and then they touch a fellow child…

**M: What would happen?**

R: They may contract corona from that.

**M: Okay, I am very grateful for your responses and time. Do you have a question for us?**

R: No.

**M: Okay, thank you very much.**

R: Thank you.

**[End of audio]**
