# Supplementary material for: Mapping social accountability actors and networks and their roles in water, sanitation and hygiene (WASH) in childcare centres within Nairobi’s informal settlements: A governance diaries approach
Source: PLoS One. 2022 Nov 15;17(11):e0275491. doi: 10.1371/journal.pone.0275491 (PMC9665391; doi:10.1371/journal.pone.0275491)
Supplement: S1 File — (ZIP) [file pone.0275491.s001.zip › Anonymized Transcripts Plos (SAM)_Oct 2022/IDI 2_Study site B (Male Respondent).docx]

**PARTICIPANT: Parent of Child attending childcare centre**

**AREA: Study site B**

**Key**

**M: Moderator**

R: Respondent

**M: Hallo and welcome to today’s IDI on social accountability on WASH. We are in Viwandani. I’ll be your moderator, FS1 and our participant is a parent with a child in the daycare center. So the first question is, when you take your child to the daycare center, whose responsibility is it to ensure that the child has access to clean drinking water?**

R: It is the responsibility of the daycare owner.

**M: What should she do?**

R: She should have water for the children’s hand washing and to make sure that the toilet is cleaned and the environment is clean.

**M: Is there anyone else accountable?**

R: Even the parent is accountable.

**M: How?**

R: Because the parent should make sure that the water the child drinks is treated. She should go to the daycare and confirm that.

**M: And whose responsibility is it to ensure that the child has access to the toilet?**

R: The daycare owner.

**M: And what about hand washing?**

R: The daycare owner.

**M: Is there anyone else apart from the daycare owner and the parent who is responsible?**

R: Even the government can be involved.

**M: How?**

R: Because as a government there are CHVs that do collect data in the community and we do see them check whether there are hand washing facilities available.

**M: Do the CHVs always go to the daycares?**

R: Yes, they do visit them.

**M: Okay, so we have the daycare owner, parent CHV and the government. Who is the most accountable person to ensure that the children have access to sanitation and water services?**

R: The daycare owner.

**M: Why?**

R: Because it’s her business and job.

**M: And to what level should the daycare owners, parents and CHVs be accountable?**

R: Not more than the daycare owner.

**M: So what level of accountability should the daycare owner have?**

R: She should be 100% accountable.

**M: Why?**

R: Because if a disease outbreak starts in her daycare then she will be held accountable by the government and she will also lose clients coming to her daycare if she doesn’t maintain hygiene.

**M: So if you compare how you are involved in the WASH of your child with how the other parents are involved, what can you say about that?**

R: I am perfect as compared to the others because I do personally visit the daycare centers and check the hygiene situation.

**M: So if you were given an option to transfer your child from the current daycare, would you accept that?**

R: I wouldn’t accept.

**M: Why?**

R: Because I have assessed the current daycare and realized that it is clean and safe.

**M: Let’s now talk about hygiene; so you do take your child to the daycare in the morning and take her back in the evening, right?**

R: Mhm

**M: How can you confirm that while at the daycare your child has quality hygiene services?**

R: Sometimes I do visit. Another is if the child doesn’t have any diarrhea.

**M: And how can you confirm that while at the daycare your child has quality water for drinking?**

R: I am not 100% sure but the child doesn’t fall sick. That’s what makes me know that the water is quality.

**M: And what confirms to you that while at the daycare the child has access to quality sanitation services?**

R: My child doesn’t use the toilet. She uses potty and it is always clean.

**M: And when a child is at the daycare and they want to use the potty, how can they report that to the daycare owner? Or how will the daycare owner know that the child wants to use the potty?**

R: The daycare owner knows – after the children are fed I do see them all being given potties and asked to have their long calls and they are then put to sit there.

**M: So there are times when the daycare owner gives them the potties?**

R: Yes.

**M: Sometimes you may feel pressed after a short time, how will they tell the daycare owner that they want to go for a long call again or how can the daycare owner know?**

R: To be honest I don’t know how they tell them because when I started taking my child she was too young though right now she has started talking so she can say it.

**M: So she can say it.**

R: Yes, but when she was younger I don’t know how they were communicating with the daycare owner.

**M: And how does the daycare owner take that information when the child tells her that she wants to go for a long call?**

R: She doesn’t take it negatively.

**M: What does she always do?**

R: She goes and shows her – the potty is always placed in a separate place, so she shows her where to go.

**M: Okay, and does she always tell you whenever she wants to go for a long call?**

R: Yes.

**M: Okay, and let’s say the child is at the daycare and she wants to drink water, how does she tell the daycare owner and how does she tell you while at home?**

R: Nowadays she tells me that she wants to drink water.

**M: And what if she is at the daycare?**

R: Even when she is at the daycare she tells the daycare owner.

**M: And how does the daycare owner always take that or what does she always do?**

R: She gives her water.

**M: Let’s say for example a child has challenges using the potty or the toilet or maybe they cannot reach the hand washing tap, what do you always do?**

R: For those who don’t use potties – by the way even my child I used to take her before she started using potty. She was very young. So she used to have diapers. I would pack some diapers for her to change.

**M: And when the child wants to wash her hands and the hand washing station maybe is too high for them to reach…**

R: The teacher will wash her hands.

**M: And what do you always do?**

R: I do wash her hands.

**M: Is there anything else you’ve been doing?**

R: Apart from washing her hands?

**M: Yes, washing her hands or giving her diapers?**

R: The daycare owner or me?

**M: You.**

R: I pay the daycare owner.

**M: And let’s say the child uses potty at the daycare but it is dirty or like we said, she wants to wash her hands but she cannot see any soap, who do you expect to solve that challenge?**

R: At the daycare?

**M: Yes.**

R: The daycare owner.

**M: Why?**

R: Because if the potty is dirty and the hygiene is poor then she will lose clients.

**M: Anything else you would like to add?**

R: No.

**M: That’s okay. So I would like to know, why do you think the daycare owner expects you to be responsible for your child with regards to WASH services?**

R: Because when people come together then they can succeed and hygiene is maintained.

**M: Why else would the daycare owner expect you to be responsible for your child?**

R: Because – on hand washing?

**M: Yes, why does the daycare owner expect you to wash your child’s hands and provide diapers?**

R: She expects me to provide for her to ensure that the hygiene is also maintained at home. The child can fall sick when I take her to the daycare and the daycare owner wouldn’t want that.

**M: And what does the daycare owner think your role is with regards to WASH of the child?**

R: She thinks that my role is to support her because the child doesn’t spend the night at the daycare but at home.

**M: So what roles does she expect you to support her with?**

R: Hand washing, paying her, issues to do with sanitation as well so that she can have good health.

**M: So what have you been doing with regards to sanitation, paying and hand washing of the child?**

R: I have two potties, one is at the daycare and the other I have at home. So when she is at home I make sure the potty is clean before I give it to her.

**M: What about on hand washing?**

R: I also make sure that I cut her nails then I wash her hands properly.

**M: And what about with regards to drinking water?**

R: I ensure that I boil the drinking water. Even the child does know the taste of water when it is not boiled because she will always tell me that she wants warm water. So she knows that the daycare owner should only give them warm water.

**M: So on the roles you’ve been playing like cutting the nails, hand washing and providing potties, do you think there is something you can do to help you achieve your roles better?**

R: I don’t think there is anything.

**M: So you feel okay?**

R: Yes.

**M: That’s okay. I would now like to know, whose responsibility is it to ensure that the parents follow the WASH policies and same to the daycare owners?**

R: The government.

**M: What do they do?**

R: The government should follow up to ensure that the policies are followed.

**M: And do they always do that?**

R: They follow up but I don’t think it helps.

**M: Okay, apart from the government following up, is there anyone else?**

R: The NGOs.

**M: What should they do?**

R: They also support.

**M: How?**

R: They do provide – I saw them provide hand washing containers.

**M: Thank you. And I would like to know what the policymakers do to ensure that when the children are at the daycare they can access sanitation services, hygiene or water services as well. What do they do to ensure that the services are available?**

R: Are you talking about the NGOs or government stakeholders?

**M: The government stakeholders mostly.**

R: I don’t think they support in any way.

**M: And what do the NGOs do?**

R: NGOs support because I saw them distribute containers. Like Red Cross helped provide the containers.

**M: Apart from Red Cross is there anyone else?**

R: I only saw Red Cross.

**M: Okay, let’s now talk about the daycare managers and teachers; so what do the daycare owners do for you as a parent to trust their services with regards to water, sanitation and hygiene at the daycare?**

R: I trusted them because whenever my child came home and I gave her a potty that wasn’t clean, she would reject it and wouldn’t totally use the potty if it wasn’t clean. So I believe that it is always clean at the daycare. Secondly, on water I trust them because whenever I give the child cold water she refuses and tells me that she wants warm water.

**M: Thank you. And what do you do to ensure that there is cooperation between you and the daycare owner or the daycare owner and the children?**

R: Between the parent…

**M: Let’s say between the parent and the daycare owner; what do they do to ensure that there is cooperation?**

R: We just communicate.

**M: How?**

R: Whenever I go to the daycare – there was a time I went to the daycare and I found that they didn’t have a mattress for the children to sleep on and I told the daycare owner that she should look for money and buy a mattress for the children so that they can sleep in a comfortable place.

**M: Okay, so one of the ways is through talking to the daycare owner face to face.**

R: Mhm

**M: Is there any other way through which the parents communicate with the daycare owners?**

R: Maybe they can make phone calls if the child isn’t feeling okay.

**M: Is there any other way?**

R: No.

**M: Thank you. And what do they do to ensure that there is transparency in their WASH service deliveries?**

R: I don’t think it’s always hidden because everything is open.

**M: What is open?**

R: I don’t see them having any secrets.

**M: For example, if a parent wants to see the toilet, are they allowed?**

R: Yes.

**M: Okay, and if a parent wants to taste the water are they allowed?**

R: They are allowed – they are allowed on everything.

**M: Thank you. And what do they do to ensure that there is equality in how the children are treated with regards to WASH?**

R: They are friendly with the children because the child may even reject you and say that they want to go to the daycare.

**M: So they are friendly?**

R: Mhm

**M: Okay, and whenever for example you tell the daycare owner that the place the children sleep is not right, how do they always take the information or what do they always do about it?**

R: They take action because the daycare owner I told took action and bought a mattress. The child even had a TV brought for them.

**M: And what do they do to ensure that the items they ask from the parents like tissues or soaps are affordable?**

R: They tell every parent to bring the items and we do provide them.

**M: Okay, how many do you always take?**

R: Tissues?

**M: Mhm**

R: We used to take three.

**M: After how long?**

R: A month.

**M: And is that affordable?**

R: Yes.

**M: Okay, and what do they do to ensure that the WASH services they provide are quality?**

R: I just see them try – when it comes to water right now we have a challenge because it is brought by the truck but I do see them treat the water. So it is quality.

**M: And what do they do when it comes to potty?**

R: They also clean the potties using omo – I also saw they bought harpic.

**M: And what about on hand washing?**

R: On hand washing right now there are containers but before we didn’t have hand washing containers.

**M: How long ago was that?**

R: When we first took our children they would always wash their hands in a basin. So when corona came they were trained on washing the hands using running water and the basin was abolished.

**M: Thank you. And what do they do to ensure that the WASH services are available at the daycare?**

R: They just talk to the parents because it is the responsibility of the parent to take potty to the daycare.

**M: That’s good. Let’s now talk about you as parents; what do you do to adhere to the payment on tissue and soap that you are asked for?**

R: We just adhere because you know, if you don’t pay then you will not get a good daycare. So you have to adhere and pay as required on time.

**M: And what do you do to follow up on the water, sanitation and hygiene services at the daycare to ensure that it is quality?**

R: In my free time I do visit the daycare and I take the child some fruit to the child while I check to see whether they are doing okay.

**M: Thank you. Is there anything else?**

R: No, I only visit the daycare.

**M: Okay, and what do you do to ensure that you are involved in the WASH service provision?**

R: Me?

**M: Mhm**

R: Because the child is mine and if she falls sick then she will be my responsibility. So I have to ensure that she is okay.

**M: Okay, and what do you do to ensure that the WASH services are used the right way at the daycare like potties, water and hand washing?**

R: I do also talk to my fellow parents and they tell me – sometimes I ask my fellow parents what they think of the daycare and they say that it is okay. They also tell me about their experiences at the daycare.

**M: That’s good. I would like to know what the government – you told me that you haven’t seen the government support in any way…**

R: The government is there because they do send the CHVs who will go round taking data.

**M: Apart from sending the CHVs, what else do the policymakers do to enhance the WASH services at the daycare centers?**

R: Right we have water provided by NMS.

**M: Are they brought to the daycare centers or to the community?**

R: It is brought to the community although the daycares that are in the interior part of the community cannot access the water.

**M: So what do you always do to get water?**

R: Maybe you would have to queue like the rest.

**M: And what would you recommend the policymakers do?**

R: I would recommend that the daycare centers be recognized by the government so that they can be given priority whenever they are fetching water and hence they won’t have to queue with the rest.

**M: And apart from providing water, what else do they do with regards to sanitation, water and hygiene? You’ve told me that there is an NMS water truck; is there anything else they do?**

R: The government?

**M: Yes, or the policymakers?**

R: I don’t think so. Maybe the NGOs are the ones that provide containers.

**M: What do the NGOs do?**

R: They provide containers.

**M: They provide containers?**

R: Mhm

**M: Okay, and what do the daycare owners do to improve WASH services in the daycare centers?**

R: They employee more workers. There is one that is charged with cleaning the toilet.

**M: Is there anything else they do?**

R: Sometimes I do see them cooperate with the other NGOs because I saw our daycare was provided with the children’s bicycles and such things. They also work hard to look for people to support them.

**M: That’s good. And what would you recommend the service providers to do to help improve the WASH services in the daycare?**

R: Maybe if possible, they should have water a special water point so that they won’t have to queue for the water supplied by the NMS; if they can have a special water point to the daycare centers that would be very good.

**M: And what do you do as parents to help improve the WASH services at the daycare centers?**

R: What we do is when the parents go to the facility and they are given water treatment tablets, maybe they are 10, you can take two to the daycare center to be used to treat water.

**M: Thank you very much for your responses. And what would you recommend the other parents to do to help improve WASH services in the daycare centers?**

R: I would recommend that we cooperate with the other parents for the services to improve.

**M: Thank you for your responses. I would like to know, do you have any special model or strategy on WASH that you would recommend and that you think can help improve WASH services in the daycare center?**

R: The model I would recommend is that they can get donors…

**M: Who can get donors?**

R: The daycare owners should get more donors then the services can improve.

**M: Why do you say so?**

R: Because sometimes money can improve everything. Sometimes she wants to buy some toys for the children but she doesn’t have the money. So a donor can support her.

**M: So that’s the toys for the children.**

R: Yes.

**M: And when talk about sanitation, water and hygiene; what model…**

R: Also if they get donors with regards to that such that they get a water point for themselves then it can take long. So if they get donors then they can be supported in setting up the water point and even construction of the toilets because the toilet that they use is public. So that one is used by everyone – so if the children can have their specific toilet then that can be good.

**M: And how do you think that this model will affect the health of the children?**

R: It will improve.

**M: Thank you for your opinion. We know that corona came with a lot of issues. So how did corona affect the children’s hand washing at the daycare?**

R: The effect?

**M: Yes, or what changes did it bring with regards to hand washing?**

R: It lead to changes by the way one being that when corona came, hygiene was improved because they were trained on hand washing using running water. So the number of children also reduced in the daycare because the parents feared contracting corona. Many parents also lost their jobs and businesses and had to be in the house with their children. So the daycare owner also lost their clients.

**M: So corona made the children wash their hands frequently?**

R: Yes.

**M: And what changes did it bring with regards to drinking water at the daycare?**

R: Drinking water, even before corona came they were drinking clean water. They used to treat and boil. By the way the woman also makes porridge for the children.

**M: So even before corona they were using warm and treated water?**

R: The water for hand washing was the challenge.

**M: So there isn’t a change with regards to water?**

R: On water maybe the change is that right now there is water shortage.

**M: So during corona water is not easily available?**

R: Yes.

**M: Okay, and did corona bring any changes on sanitation at the daycare center?**

R: No, the change was only that the children in the daycare reduced. They were very few.

**M: And how did that affect sanitation services?**

R: The sanitation services were just okay.

**M: It was just okay?**

R: Yes, but they used to use the Fresh life toilet and they would visit frequently and provide soap and also check the toilet. I used to see them come.

**M: Okay, so that’s during corona?**

R: Yes. They used to come frequently.

**M: And before corona came they weren’t visiting frequently?**

R: They were visiting but not frequently and they weren’t also providing soap the same way they provided during corona.

**M: Thank you very much for your responses. I think we have come to the end of our discussion unless you have something you would like to tell me.**

R: I don’t have any comment but I have a question; after this research how will you help the parents with children in the daycare centers?

**M: So you are a parent, right?**

R: Mhm

**M: We are also talking to the daycare owners to get their opinions. So we’ll take all of these opinions and take them to the government or policymakers and tell them that the daycare parents are saying this about WASH and the daycare owners are also saying this and they all recommend support at this place. So they are the ones up there and so we’ll take them the report on what you think so that in case they will provide any support they will do it in the daycare centers and I know that if support is provided to the daycare centers then the community will have benefited because your child will be going to the daycare and if they fall sick then you would be responsible. So that’s what we are doing. The report will be taken to the government and then it will bring some changes.**

R: That’s okay.

**M: Thank you very much for your time.**

R: Thank you.

**M: So the interview has ended at 2:07pm.**

[End of audio]
