# Supplementary material for: Mapping social accountability actors and networks and their roles in water, sanitation and hygiene (WASH) in childcare centres within Nairobi’s informal settlements: A governance diaries approach
Source: PLoS One. 2022 Nov 15;17(11):e0275491. doi: 10.1371/journal.pone.0275491 (PMC9665391; doi:10.1371/journal.pone.0275491)
Supplement: S1 File — (ZIP) [file pone.0275491.s001.zip › Anonymized Transcripts Plos (SAM)_Oct 2022/IDI 3_Study site B (Female Respondent).docx]

**PARTICIPANT: Day care owner**

**AREA: Study site B**

**Key**

**M: Moderator**

R: Respondent

**M: Hello and welcome to today’s IDI and social accountability of WASH. We are in Viwandani. I will be your moderator FS2. My participant is a daycare owner. Today being on Tuesday 29^th^ March 2022. The starting time of the interview is 11:32am. Welcome. Just as I have told you that our main topic of discussion is WASH; and in WASH we shall be discussing each of the elements one by one. So maybe let’s start with hand washing at the daycare, whose responsibility is it to ensure that those children wash their hands thoroughly with water and soap?**

R: It is responsibility.

**M: In what way?**

R: If I want to feed them, I wash their hands; if they touch the ground I also wash their hands, while changing their diapers I have to wipe them.

**M: Is there any other person with the responsibility to ensure that the children wash their hands apart from you?**

R: The parents.

**M: In what way?**

R: When she comes to pick the child she has to wash her hands first before taking the child.

**M: Okay is there any other person?**

R: No one else comes.

**M: Okay, it’s only them?**

R: Yeah.

**M: As a daycare owner how can you know that a child has thoroughly washed their hands with water and soap? For example, that quality hygiene, how can you know that a child has ensured quality hygiene?**

R: Because they are young children I have to personally wash their hands to ensure that they are clean and that their hands are thoroughly washed with water and soap. Because they are young children you can’t let them wash their hands by themselves. You have to wash their hands personally.

**M: When the parents come how can they know that the child’s hands have been washed clean?**

R: They will just take a look and notice that the child is clean and has no dirt on the face. Their hands have been washed and they are clean.

**M: Thank you. Now I would like to know—you’ve told me that the parent will just come and notice how the child looks. I would like to know, when the child is at the daycare how are they supposed to report that maybe they haven’t washed their hands?**

R: A child?

**M: Yeah.**

R: Sometimes when the child touches dirt they take a look at their hands and start crying so that you can wash them. For example, if this one touches dirt you will see him showing you.

**M: He shows you that he has dirt?**

R: Yeah.

**M: How about those that can talk.**

R: Those that can talk will tell you that they want to wash their hands and eat. There are some that are old enough to talk and their parents leave them here while they go to work.

**M: Is there another way?**

R: There is no other way.

**M: Thank you. When they cry for you wash their hands, how do you always respond to them?**

R: Theirs?

**M: When the child cries for their hands to be washed, how to do you respond or what do you do about it?**

R: I just wash them and wipe them. You will just realize that the child needs to be cleaned. The child likes hygiene.

**M: So when the child is at the daycare they will cry for them to be washed. And when they are at home how will they give such a report to their parents?**

R: To their parents?

**M: Yeah.**

R: The parent will just notice that the child is dirty or the child will not touch or hold anything that they give to them.

**M: Thank you. Now let’s talk about drinking water; whose responsibility is it to ensure that the child gets access to drinking water at the daycare?**

R: It is my responsibility.

**M: Okay…**

R: Yeah because sometimes these children when their mothers bring water sometimes they don’t boil it. So I do boil the water. I use stove to boil enough water for the children. Some can come without water. A mother might just bring the child without water, you know when a child feeds they must drink water. So if you feed them you have to give them water to drink.

**M: So there is you and also the mother that provides them with water. Is there any other person?**

R: There is no one else.

**M: How is a child supposed to report when they need drinking water?**

R: When they want water?

**M: Yeah.**

R: You’ll just see them ask for it when they see it. Those that can’t talk will show you by pointing at the water while those that can talk will just go and get the water themselves.

**M: When the child points at the water, how do you respond to them?**

R: I give it to them because they want it.

**M: And when they are at home how will they report?**

R: When they are at home they will show their parents that they want to drink water. Or they will cry when they see someone drinking the water while they don’t. Or if they see the other children drinking water they will cry for them also to be given water.

**M: Okay now we are done with water and hand washing. Toilets; whose responsibility is it at the daycare to ensure that the child goes to the toilet?**

R: It is my responsibility.

**M: Okay…**

R: Yeah because the younger ones must—those that use the potty must have the potty, the younger ones that use diapers you much check them to see if they have fecal wastes and those that can use the toilets just go to the toilet.

**M: Is there any other person with the same responsibility?**

R: There is no one else. It’s just me.

**M: When a child is at the daycare how are they supposed to report that they want to use the toilets or maybe they want a diaper change?**

R: A younger child that can’t talk will cry or become restless then you’ll realize that they need a change of diaper.

**M: What about a child that can talk?**

R: Those that can talk will just tell you that they want to defecate.

**M: And how do you respond when they tell you that?**

R: You give them the potty or you take them to the toilet and you ensure that they use toilet paper to wipe themselves and you wipe those who can’t wipe themselves. For the younger ones you ensure that their mothers bring wipes with them because you have no time of warming water every time.

**M: As a daycare owner, how will you know that a child uses a safe toilet or a safe potty or a safe diaper?**

R: Sometimes the mother may bring the child to the daycare with diapers worth 10 shillings and a child that last with such a diaper for more than 2 hours. The child might get a diaper rash after urinating in the diaper, therefore it’s my duty to ensure that the mother brings the right diapers to the daycare. I also make sure that the potty is clean enough and aired in the sun to avoid infections on the child.

**M: And how do you know when they are not cleaned properly.**

R: When a child sits on it you’ll notice that they have rashes after three within three to four days; you’ll know it was not cleaned properly.

**M: Rashes on which part?**

R: On the buttocks.

**M: Okay. Is there any other way to ensure that the child gets access to a hygienic toilet?**

R: There is no other way. You just have to ensure that the potty is washed and cleaned properly.

**M: How do you ensure that a child gets clean drinking water?**

R: We boil the drinking water; I make sure that they drink boiled water because if you give a child water that is not boiled to drink the child will suffer from diarrhea because the water is a recycled type.

**M: And how will a parent know that their child got clean drinking water at the daycare?**

R: The parent will know because if a child is brought to the daycare, some daycare owners don’t provide boiled water. So if the child starts to diarrhea or to vomit the parent will just realize that the child doesn’t get access to clean drinking water or proper hand wash at the daycare.

**M: Thank you for your impressive opinions. So you’ve told me that those who are responsible for water and toilet services are mostly the daycare owner and the parent?**

R: Yeah, mostly the daycare owner.

**M: Why.**

R: You know it’s the daycare owner that spends most of the day with the child. The mother will just come for the child in the evening.

**M: Thank you. To what extent should the daycare owner and the parent take the responsibility on matters to do with WASH?**

R: The person that should be more responsible is that person that spends most of the time with the child. This is because the mother might tell you to bath the child for her because she comes for the child late in the evening. They bring you clothes to change for the child. She only comes for the child to go to sleep.

**M: So on the matters of WASH, are you supposed to be less responsible, averagely responsible, highly responsible or extremely responsible?**

R: Extremely responsible.

**M: Why?**

R: Because hygiene is very important. And without hygiene you can’t live because how are you going to live with the children if you are not hygienic. You have to make sure that everything is washed and cleaned.

**M: Thank you. So how do you compare your way of taking care of the children in your daycare and that of the other daycare owners?**

R: Personally I can say that I do ensure maximum hygiene in my daycare.

**M: If you compare yourself to the other owners?**

R: If I compare myself with the other owners. Because when the mother comes she finds her child clean and unhurt. I take good care of them.

**M: On matters to do with toilets; how do you compare yourself to the other daycare owners? Toilets, potty and diapers, how do you compare yourself with the other daycare owners?**

R: For instance, I can’t leave the used diapers in the open for the children to play with; I’ll make sure that I put it somewhere safe. I also ensure that the toilets are clean so that children don’t get infections.

**M: Thank you very much. Now that the daycare owners and the parents are the most responsible though you’ve told me that the daycare owners are the most responsible. I would like to know who has the biggest influence when it comes to WASH.**

R: It’s the daycare owner. This is because the child is daily brought to the daycare and therefore it’s the responsibility of the daycare owner to ensure that the child’s hands are washed, the child has clean water to drink and also to ensure that the child is safe. It’s therefore the duty of the daycare owner to be more careful. The child’s mother trusts the daycare owner with the child’s safety because she only comes for the child late in the evening.

**M: Okay. In case a child has a challenge with matters concerning WASH; maybe a child doesn’t know how to use a potty or may be a child doesn’t know how to wash their hands or maybe they are unable to access the hand washing container, what can you do about it or what can you advise the child to do?**

R: I personally wash their hands because they can’t wash them by themselves especially the younger ones like this one.

**M: What about matters to do with toilets?**

R: I make sure the toilets are properly cleaned using a disinfectant. I ensure that I remind the children who use the toilets to use them appropriately and to wash their hands afterwards.

**M: So that’s how you’ve been doing it?**

R: That’s how I have been doing it even now.

**M: And who else do you expect to take such responsibilities?**

R: I expect no one else but myself to take the responsibilities.

**M: why?**

R: Because I’m the one who spends most of the time with the children, so I don’t expect someone else to come and do the cleaning. I do it myself because even the parents expect to find their children in a clean environment.

**M: Why do the parents expect to find their children in a clean environment? Why do they expect you to provide their children with clean potties? Why do they expect you to do all that?**

R: I’m supposed to do all that because I don’t want any complaints from the parents. If I don’t do all that then they won’t bring their children to the daycare again.

**M: Why do you think so?**

R: Because even me, personally I can’t allow my child to be in unhygienic place because that will cause infection to the child. When it comes to children, hygiene is very important.

**M: When it comes to matters concerning WASH; toilets, drinking water and hand washing. What are the parents’ expectations on your responsibilities on WASH?**

R: The parents expect that my responsibilities include giving the children clean drinking water after feeding them, wiping them after they defecate and washing their hands.

**M: And how have you been carrying out the responsibilities?**

R: I have been making sure that I do all the necessary by washing their hands, wiping them after they defecate, cleaning them. You know I child of this age will cry when they need a diaper change. I’ll make sure that I clean the child to ensure that they are comfortable enough.

**M: What are the possible measures that you can take to improve on carrying out your responsibilities?**

R: For instance, a parent might bring their child to the daycare without any food such as even porridge; if we find a sponsor that can provide the children with food then the children will be happy and they daycare will improve. Even the parents will be happy because they will be at peace knowing that their children cannot go hungry at the daycare even if they are not in a position to provide for them.

**M: Back to matters concerning toilets; what are the measures that you can take to improve on your responsibilities on matters such as potty, diapers and hand washing to ensure that you serve the children better than you are serving them now?**

R: For the young children who lack diapers and cannot use the potties I would buy them diapers if I was in a position to do so.

**M: Any other measures?**

R: No other measures.

**M: And who does the supervision to ensure that the daycare owner abides by the rules and regulations of WASH?**

R: No one. It’s upon me to ensure that all the rules and regulations of WASH are followed because no one will come to ask you why a child hasn’t washed their hands. It’s upon you to make sure that the rules are followed for there to be a clean toilet, the children’s hands are washed and the children are clean.

**M: If you don’t clean the toilets nor wash their hands, will anybody question you? Or is there someone that will ensure that you wash their hands?**

R: No one will. Though if the parent comes they may notice the dirty toilets and the dirty hands and they will ask why and complaining. In the end, they will stop bringing the children to the daycare.

**M: Okay. There are these people called policy makers who are government employees; I would like to know how they have participated in ensuring that there is availability of WASH services in the daycare facilities.**

R: Sometimes they visit the daycare facilities to do a research on the activities that take place in the daycare facilities. They ask how the children are being handled; they ask if the children wash their hands.

**M: What if they find out that the WASH services are not available?**

R: Then it’s upon the daycare owner to find the solution.

**M: How?**

R: For instance, it’s upon the daycare owner to provide diapers to the less fortunate child or wash their nappies and air them to dry and to reuse them.

**M: Apart from inspection, what other measures are taken by the policy makers to ensure that the children have access to the WASH services?**

R: None.

**M: Do they provide you with any materials to assist you with the provision of WASH services?**

R: No materials.

**M: As a daycare owner, how do you ensure that the parents trust you with their children’s safety?**

R: A parent might take notice of how their child is being handled. Maybe the child was brought to the daycare when malnourished, you feed them a balanced diet and their condition improves. After two to three months they realize that the child has added some weight and that’s how the daycare owner gains the parent’s trust especially when the daycare owner ensures that the children are well fed and there is no left over. Some daycare owners don’t feed the children well because the work is hectic; it’s one daycare owner against a number of children.

**M: So that’s about feeding. What about WASH services? What measures do you take to ensure that the parents trust you with WASH services provision to their children?**

R: They will see that the toilets are clean, the children’s hands are clean, the children are not suffering from diarrhea due to failure to wash their hands and the children are clean. That’s how they come to trust you.

**M: How do you ensure proper communication between you and the parents?**

R: You have to tell them the requirements in order to take good care of the children because some parents will bring the children without diapers—you have to tell them for instance if you need soap you have to tell the parents. You have to tell them that you need disinfectants for washing the toilets. You also need to discuss with them what’s needed for the child’s well-being.

**M: What means of communication do you use with the parents?**

R: I just call them. When the parent comes for the child I discuss with them what is needed to ensure the well-being of the child.

**M: Is there any other means of communication that you use?**

R: There is no other means because that is the only way that you can reach them.

**M: Okay. And how do you ensure transparency in the provision of the WASH services?**

R: They just find everything that they need for example water for hand washing, boiled water for drinking, clean utensils, all is provided.

**M: How do you ensure equity among the children when it comes to provision of WASH services?**

R: You just make sure that all the children get access to all the WASH services. You make sure that you don’t discriminate any of the children and that you treat them equally.

**M: Let’s go back to communication; is there any means of communication between the daycare owner and the children?**

R: There is no means. You just talk to the children directly.

**M: If a parent comes and gives some instructions on how to handle their child or maybe for example a policy holder tells you that your toilet is not clean, how do you handle such cases?**

R: You just listen and take their complaints or their instructions into consideration and ensure that you make adjustments and improve on them.

**M: Does your facility require for necessities such as toilet papers and diapers from parents?**

R: Yeah the parents are required to provide things like diapers because I’m also not in a position to buy them. I do tell them to provide every young child with the jumbo packet of diapers per month.

**M: I know for a fact that at some point the parents are required to pay for the provision of the WASH services. How do you make sure that the charges are affordable for the parents?**

R: For instance, you can’t charge a parent 100 shillings at the moment because they are also financially unstable.

**M: So?**

R: Most of them will tell you that they can only afford 50 shillings.

**M: Okay. What exactly is the 100 shillings for?**

R: For instance, handling a young child is more work than the rest and so you have to charge their parents more; that is maybe 100 shillings. The parents will tell you that they can’t afford that amount and they will end up paying 50 shillings only or maybe 30 shillings. You’ll just have to sympathize with them and accept the little they can provide.

**M: What about the situation where the diapers are too expensive for some parents to afford?**

R: You just tell them to make sure that they bring the diapers.

**M: What measures do you take to ensure that you provide the children with quality WASH services?**

R: We ensure hygiene in every aspect. We ensure that the children are clean, their hands are washed, the toilets are clean, the drinking water in boiled and stored in a closed container.

**M: And how do you ensure that their hands are thoroughly washed?**

R: You have to ensure that they have access to the hand washing materials such as soap and water.

**M: How do you ensure that every child has access to the WASH services? How do you ensure availability of the WASH services to the children?**

R: The WASH services are just readily available within the facility.

**M: Is there any other way?**

R: There is none.

**M: Let’s talk about the parents; how do they make sure that they pay the 50 shillings that you charge them for the diapers and the tissue papers?**

R: They just do the work – what else can they do?

**M: Okay, and do most of them pay at the required time?**

R: No. some don’t even pay. Someone can bring you a child and tell you that they would pay at the end of the month and she doesn’t pay. Maybe she has a debt of 600 or 400. So she will just decide not to bring the child anymore. She will transfer the child to a different daycare. They will just decide to transfer – maybe they feel that you let them bring a child and they got to a debt of 2,000 and she can’t pay it.

**M: And what do you do to prevent such like things?**

R: I do tell them sometimes that it shouldn’t get to Saturday because they always leave then. Even some people will transfer to the other daycare on a Sunday. So they do move too different daycares because it’s not easy.

**M: Thank you. And what do the parents do to ensure that they follow up on the WASH services to ensure that they are of quality?**

R: They do come. Like I told you, they have to come and check that the toilet is clean. For example, if you don’t clean the toilet and they see it they will then tell you because as a parent you cannot take your child to a dirty environment because even when you are at work you will not be happy to know that your child is in a dirty environment.

**M: And what do they do to ensure that they are involved in the provision of the tissues you ask from them and that they are involved in the management of the WASH services in the daycare?**

R: They will make sure that everything is available.

**M: Is there any other thing?**

R: No.

**M: And what do they do to ensure that they are accountable on the use of the services?**

R: Let’s say, if a parent brings whitewash soap or diapers and it doesn’t last a month, she will start saying that the diapers were used on some other child. So they do complain. So they do make sure that the diapers last for a month. If you misuse it then they won’t accept that it’s finished.

**M: So you’ve said that they do complain.**

R: They do complain.

**M: What methods do they use to raise their concerns?**

R: They do ask themselves why the diapers don’t last for a month at the daycare.

**M: So who do they always complain to?**

R: They complain to me.

**M: And if they complain to you and you don’t change, where else can they go and complain?**

R: They cannot complain anywhere else like the chairman because they are not involved. So you have to make sure you complain to the right person. It’s very tough.

**M: So what do the parents do to ensure that the WASH services are improved at the daycare?**

R: They will come and ask me whether I have boiled the water or I have charcoal to boil the water. If I don’t have they will volunteer to buy so that they can boil the water so that the children can drink safe water. So they will just help. They do help.

**M: They help?**

R: They do help.

**M: And what would you recommend to the parents to help improve these WASH services?**

R: We should have good relationship with them because you cannot take care of a child if you don’t have a good relationship – it won’t be possible because then if you tell them that the potty is broken then she won’t bring a new one – because when you bring a child to the daycare you have to bring a potty as well. Sometimes you even tell them that they should bring three potties.

**M: Is there anything else you would recommend to the parents?**

R: Sometimes I do recommend to them that we both maintain hygiene – not just myself – we should do it together.

**M: Is there anything else?**

R: No.

**M: Lets now move to you as a service provider; what do you do to ensure that you improve the WASH services at the daycare center?**

R: We consult. For example, when it comes to the toilet I tell them to advise their older children not to use the toilet wrongly. Sometimes I do call them and we decide to do general cleaning and every parent will wash a blanket and make sure that we even clean the walls because I cannot do everything alone or else I would suffer. So they come and we clean even the walls of the toilet and the doors because the children would always touch them when they open the door.

**M: And what happens to the children who don’t attend that cleaning?**

R: They will have to come because they will be bringing their child. So they will have to participate.

**M: Is there anything else you do to improve the WASH services?**

R: No, we just maintain hygiene.

**M: And what would you recommend to the other service providers to help improve the WASH services at the daycare centers?**

R: I would recommend that people maintain hygiene in every daycare center and they wash their hands. If they don’t have hand washing stations they should buy them. The young children should have their hands washed and they should have their hands washed using soap and water in order to maintain hygiene.

**M: Anything else?**

R: No.

**M: Thank you. And what would you recommend the policymakers to do to help improve the WASH services in the daycare centers?**

R: Sometimes they can also bring the hand washing stations for the children. Also they can buy and provide more potties to the daycare center. If you only have two mattresses, they will just be provided.

**M: So what would you recommend the policymakers do with regards to sanitation, hand washing and water for the services to be improved at the daycare centers?**

R: It’s just about cooperating to make sure that hygiene is maintained everywhere.

**M: And what can you recommend to the policymakers to do so as to improve the WASH services?**

R: It is just cooperation.

**M: Who should cooperate?**

R: The policymakers and the daycare owner.

**M: Thank you. And do you have a strategy or accountability model that you know that if you use then it can help improve WASH services at the daycare?**

R: For example, if we have hand washing facilities and everything else then hygiene will be maintained and WASH services will be improved.

**M: Where will those facilities come from?**

R: We buy.

**M: Who will buy them?**

R: We will.

**M: You as?**

R: The daycare owners.

**M: The daycare owners?**

R: Yes, we’ll just buy.

**M: So you buy them so that you can maintain hygiene?**

R: Yes.

**M: Okay, so if the facilities are made available and hygiene is improved, how will that affect the health of the children?**

R: It will not affect the health of the children because you will have maintained hygiene. You know as I said, hygiene is essential because if the child doesn’t wash her hands she will put the hands in the mouth and develop diarrhea. The child won’t know that the hands are dirty, so she will just touch the diapers.

**M: And how will purchasing these facilities affect their health?**

R: It will improve; if these facilities are purchased then their health will be improved.

**M: Thank you. So I know that corona brought a lot of changes in the daycares.**

R: Very much.

**M: So what changes did it bring with regards to hand washing at the daycare centers?**

R: It brought about many changes because people could wash their hands all the time and you have to ensure that the children wash their hands all the times.

**M: So that’s a change that came about on hand washing?**

R: Yes.

**M: Is there any other difference?**

R: No.

**M: And what changes did it bring with regards to drinking water at the daycare?**

R: Drinking water?

**M: Mhm**

R: None.

**M: So did you say that you do have water shortages or is it always available?**

R: We do have water shortages like now it’s been two weeks since we had water.

**M: And if you compare the water shortages during corona and before corona; can you say that they are more common now or before?**

R: Before corona we used to have water shortages but when corona started we didn’t have a lot of water shortages because people have to maintain hygiene. Even the government through NMS provided water to the community.

**M: So what about the quality of water before corona and during corona, is there any difference?**

R: There is a difference because for example before corona came water used to come through sewer lines but when they assessed the community, water was removed from the sewer line.

**M: So that happened during corona?**

R: Yes, it happened during corona because you find that the pipes no longer pass through sewer lines.

**M: Thank you. And what changes did corona bring with regards to sanitation at the daycare centers?**

R: You know sometimes because weren’t maintaining hygiene.

**M: So people only maintain hygiene during corona?**

R: Yes.

**M: And is it hygiene in relation to sanitation or hand washing?**

R: Hand washing and sanitation.

**M: Is there any other change that corona brought at the daycare?**

R: No.

**M: Okay, so your child goes to your daycare?**

R: Yes.

**M: I would like to know, if you are given an option to take your child to a different daycare, would you accept?**

R: I cannot accept.

**M: Why?**

R: Because when I am with her in the daycare I maintain hygiene and I will also be happy to take care of the others because my child is also there.

**M: Thank you very much for your time and responses. We have come to end of our interview unless you have a comment.**

R: No, I don’t have a comment.

**M: Okay, thank you. So our interview has ended at 12:12pm.**

[End of audio]
