# Supplementary material for: Mapping social accountability actors and networks and their roles in water, sanitation and hygiene (WASH) in childcare centres within Nairobi’s informal settlements: A governance diaries approach
Source: PLoS One. 2022 Nov 15;17(11):e0275491. doi: 10.1371/journal.pone.0275491 (PMC9665391; doi:10.1371/journal.pone.0275491)
Supplement: S1 File — (ZIP) [file pone.0275491.s001.zip › Anonymized Transcripts Plos (SAM)_Oct 2022/Reflection_Study site A.docx]

**Round One: Reflection on Respondent 15 (Study site A)**

Our fifteenth participant to interview was a lady Child Care Center Owner and we arrived after lunch time while the babies were already asleep, so we had ample time to carry out our interview. She received us well because she had been recruited by our mobilizer and was present in the initial group introductory meeting at our site offices and we had also visited her the previous day but the timing was all wrong, she seemed fatigued and she advised us to visit her any day after lunch time, when the babies have already eaten and are asleep, an advice we heeded to, on this visit.

From the on-set, beddings, sleeping and storage space seemed to be a major issue and the only beddings I saw were the bed-sheet covered mattresses on the clean carpeted floor, that the children shared as they had their siesta, since there were no beds. The mattresses were laid on the floor horizontally while the children were positioned vertically so as to accommodate all of them in one room accessible from the main room, which had items like books and other stationery, diaper packs, tissue paper rolls, babies’ bags hanging from nails pinned under the shelves and babies’ shoes on the shelves, as she does not allow anyone to enter the polyvinyl carpeted rooms with shoes. The walls to this main room had preschoolers’ educational charts hanging and cartoon characters colorfully painted on them. Upon formal introductions with one other lady in this main room and a quick view of the sleeping babies in another inner room, we followed the participant out of the main room, towards our right, up to the interview room, which seemed to double up as a class room with a black board and a storage of toys and two large water storage containers covered in blue clothing, set on the left corner of the room. Outside these rooms, along the corridor to the right were four potties arranged orderly along the wall, after the hand washing point next to the door of entry into the main room.

Her busy day starts at 4.00 am and by 6.00 am. she is usually at the center to prepare it for the day and wait for parents since the first parents start coming at 6.20 am. She has a daily schedule that she follows with the children and the activities are done according to the children’s ages.

Regarding health, the informal actors that came up were Parents, Teachers and Chemists while formal ones were Doctors. She observes Corona safety measures as she takes and records down the temperatures of the babies on arrival and she had noted that most of her babies had normal temperatures during the day but it tended to shoot up to 40 degrees while they were at home and parents would call her complaining but she would tell them that the babies were very okay during the day at the center. One baby had the same issue and it was until her mother took her to hospital, that they discovered she had Pneumonia and she got admitted into ST MARY’S hospital. The next visit will seek to find out how the health and temperatures of the babies have been since our last visit and more about this baby’s case and this hospital.

On sanitation, the next visit will probe on the center’s access to toilets and where the potty contents for the babies are deposited, do they pay for it, who does that for the center and which day of the week they get deposited.

Under education, she had a daily routine for the children according to their ages and keeping them engaged reduced the noise they made. They sing and pray at 8.30 a.m., toileting and handwashing at 9.50 am, porridge and bread eating at 10.00 am, games playing from 11.00 a. to 12.00 mid-day followed by handwashing and preparing for lunch. Feeding takes time since some children are fast while others are slow eaters. There is a 10 minutes break after lunch for potty and preparing to sleep, and on waking up, they eat a fruit and get into free choice activities as they wait for their parents and guardians to come for them. The next visit will probe on how she thinks these activities affect the children’s wellbeing.

For hygiene maintenance, she does not allow anyone to enter the center rooms wearing shoes due to dust which can affect the children’s health. She and her colleague clean the center every evening and each morning. The informal actors that emerged were CHVs, Parents and Community individuals since she had seen parents who used to bring dirty babies to the center improve on their babies’ cleanliness due to her talking to them from time to time about hygiene. CHVs were mentioned to offer a helping hand at cleaning the center sometimes but not regularly. The next visit will probe on what particular days make CHVs come to help out and if they help out in other health and wellness issues in addition to maintaining hygiene.

For water, they store it in 20 liters yellow jerry cans, black basins, two buckets, one purple and one green, that are kept orderly against a wall in one of the classrooms. More water was stored in the interview room in much larger containers covered in light blue clothing, set on the left corner of the room.

When the babies awoke, I had to release her to tend to them since she is the one who receives them, prepares them and releases them back to their parents when they arrive, therefore we successfully ended our interview.

Other observations included probes for the next visit to find out more about her challenges while running the center on top of parents failing to pay up their day care rates and whether there is a formal or informal possibility of following this up to get them to pay her, since she said most of them sell goods at the market while others are hawkers meaning they make some income compared to parents who go to try their luck at casual jobs but do not find any.

**Round Two: Reflection on Respondent 15 (Study site A)**

On this day we visited a Day care center situated at the market which is a very active and vibrant area such that along the roadside there are hawkers, grocers, food and non-food commodity vendors, shops, chemists, educational facilities, churches, motor bike stages, local brew dens, water tanks, handwashing tanks, water points, second hand household items and gargets. Our timings did not work well for us because we found the participant very busy and after a few minutes, it was clear that she would not be able to focus on our discussions because of the many interruptions requiring her attention. We faced an ethical dilemma here because although she had consented to the interview and we had scheduled our appointment, it seemed she had also not anticipated her day to be so. There were a lot of interruptions from visitors and parents coming for their babies to the extent that she could not sit still for a minute before someone demanding her attention. We could only get through her diary and the probes from our last visit.

1. **Water**

Treated water reduces diseases.

Vulnerability

She is keen on the water that parents bring for their babies and she prefers to give them treated center water instead of the water they have carried form home when she sees that it is not very clean.

**Accessibility**

Community.

**Accountability/Actors**

Parents, Teachers, CHVs.

She gets tablets for treating water and Water guard from a CHV when they are available at the public hospital at the Chief’s and the CHV also talks to them about hygiene and health.

**Probes for the next visit.**

Any changes to her hygiene and solid waste and who will manage these while she is in upcountry?

Is there a difference between her and others in accessing basic services and experiences?

What challenges does she face in accessing these services?

If she knows about access to these services in her grandchildren’s schools?

What is the role of guardians in ensuring children access these?

What are her expectations on accessing these services?

If she ever has meetings with teachers to discuss these services in schools?

What amount of water she utilizes in a day in liters?

What consumes most of her water?

How much water goes to hygiene?

If some days use more water than others and why?

Where fecal waste gets deposited by the workers?

What is her opinion on toilets?

Are services serving her interests?

Are services adequate?

What is her priority as she goes into a public hospital?

Where do the rest go for services?

What do they do about issues as a marginalized group?

What is important for her at this point?

### Round Three: Reflection on Respondent 15 (Study site A)

This Day care center is situated at the market and in front of it are second hand clothes being sold. We found all the other babies asleep apart from one child who we later confirmed that he was autistic and she was trying to feed him as he ran and jumped all over the room. From face value he looked well and joyful but when you studied him keenly, you would notice how his eyes tended to revert to the corners as he focused on you. He could not speak and when food was put in his mouth, he would pinch his nose and the Teacher would stop him from doing that. We asked why he does it and she said before his mother knew that the boy had a condition, she would force feed him by pinching his nose to force him to swallow the food. The kid ran and jumped up and down up to the end and by the time the interview was ending, he had jumped on my back already and when I joked with him that I was going away with him, he jumped down again and circled round and round. During the interview, she was open and she smiled while responding. At times we had to pause the interview to let her attend to a visitor and to the autistic child, then we would resume. She would be attentive but when the time for preparing the children for home approached, she became pre-occupied and by the end of our interview, the other babies were already up and the other teacher and already removed their bags awaiting their parents as their hot porridge cooled down on the table. From the quiet that we had coming, now voices of children could be heard and we knew it was their time for home.

Water

It is used a lot in handwashing.

Days that consume most water are:

Rainy thus muddy days

When a baby soils herself thus must be bathed

When babies are having bouts of diarrhea which require them to be bathed.

Very hot days make the children ask for a lot of drinking water frequently and thus more water is consumed.

When children are having common colds, they tend to drink more water than ordinarily.

Potties are emptied and washed thrice a day and this takes a lot of water too.

During 2 to 3 weeks of scarcity, the center buys water from vendors with water in containers riding on donkey carts move around selling water whose source is unknown and they treat it with Water Guard.

200 liters per day when water is available

160 liters per day during water scarcity

Cleaning the outside is reduced to prioritize on

Vulnerability

Dirty water flowing out of taps, smelling of sewage at times exposes the children and the center teachers to water borne diseases.

When alone, managing the center’s basic amenities and activities is a challenge.

Those not potty trained use it carelessly and when alone, it becomes a challenge to care for the babies and handle hygiene matters adequately because they cannot be left alone even for a minute as anything can happen.

Toilet sharing by people of all ages may cause infections to the users especially girls.

Marginalization

Physically challenged and Autistic children require extra care yet they pay the same rate as the rest, since the teacher does not want their parents to feel marginalized by paying more yet all parties are innocent.

Plot tenants wantonly neglect general plot hygiene and leave it all to her and her aide, who also complains of the toilet yet there is water there to pour in and a roll of tissue.

On Sundays, plot tenants and church members willingly neglect to wash the toilets that they all use and leave it to her to clean on Monday morning, making her feel marginalized and disappointed.

Accountability/Actors

Government

Village elders

Water sector staff who are supposed to come and read the water meters in the villages never do.

Parents and Teachers Community members

Plot care takers

Landlords

Equity

None since some plots can access water while others cannot.

Water comes out of taps dirty after being scarce.

None in Sanitation since all gender and ages use the same toilet.

**Other observations included the following probes for the next visit:**

Hygiene

Any changes to her hygiene?

How much water goes to hygiene?

Where are actors found?

Where do they get their authority from?

Do the actors serve her center’s interests?

Water

If some days use more water than others and why?

Where are actors found?

Where do they get their authority from?

Do the actors serve her center’s interests?

Sanitation

Any changes from the last visit?

Where are actors found?

Where do they get their authority from?

Do the actors serve her center’s interests?

**Round Four: Reflection on Respondent 15 (Study site A)**

Our fourth visit to our fifteenth participant takes us to a Day care center that is at the main market where we find our respondent comforting one baby who was not yet asleep, with the other teacher mopping the corridor of the plot. The place is calm as they welcome us with an exchange of greetings and handwashing before entering the room. Once inside, I admire the sleeping children then I look around and I wonder where the autistic boy is because he never sleeps, but runs and plays around all the time. The participant reads my thoughts and she smiles telling me that the boy is not present that day.

We commence our interview comfortably because we are in time and this particular respondent does not have a more relaxed schedule like our other Day care center owners who live nearer their centers. Her daily schedule is solid and she does not live in the same plot as the center. There are interruptions from crying babies and visitors but we manage to carry out our discussions successfully.

Health is the most important aspect of wellness because it affects how she runs the center. Hygiene is second followed by Education.

1. **Water**

As formal structures, NGO TDH brought them a handwashing water tank and Community youths are the informal structures that are the most influential players found in the community. TDH are found in their offices in Korogocho and their authority is from the government. Youths authority is traditional and they want to earn an income while the Government wants the people to get water when it is scarce. When there is a water shortage and water is brought into the village in a lorry, only the strong manage to scramble and fight to fetch it and those who are weak miss to get it. Accountability is with Nairobi water company and Nairobi city council. There is no equity because water can disappear for a whole week in the slum.

1. **Hygiene**

As informal structures, the individual and the tenants are influential while a Caretaker who maintains good security too, is another informal structure that is influential. They are found in the community and their authority is from the Landlord. The Care taker wants to collect house rent and the Landlord’s interest is in the rent money. Hygiene cannot be maintained optimally because of water scarcity, and the center uses water from other activities to clean the toilet e.g. from utensils washed, sometimes from a salon. Other tenants in the plot where the center is never help her to clean. Accountability is with the Plot care taker and the Landlord and there is no equity because other tenants do not help her to clean the plot.

1. **Sanitation**

As formal structures, a Day care Teacher is the most influential because she maintains the centers’ toilets, while another informal structure is the plot Caretaker. Formal structures are NGOs through community groups and they are found in the Community and the Chief’s office. Authority comes from the Village elders and the government, NGOs want to help the community generate income, Groups want to increase toilets and help people reduce flying toilets. People build plots without toilets. Accountability lies with the Care taker, Landlord and the Government, but there is no equity since tenants do not help her to clean the toilet.

1. **Health**

As an informal structure, a Parent is influential, while the formal structures are CHVs and the Teacher at the center, who is the most influential because she spends most of the time with the babies and she advices the parents to take babies to the hospital when they fall ill. Doctors are the formal structures that are influential. CHVs are found in the community and through phone contacts, teachers are found in the center and doctors are found in the Public hospital (Korogocho Health center). CHVs get their authority from the community. CHVs let her line up at the hospital yet she has babies waiting for her at the center. One can be told there is no medicine at the hospital and sometimes one waits for the doctors for a long time even after arriving early at the hospital. A teacher wants treatment for the baby promptly so that she can rush back to the center. A doctor’s wants to treat people and the CHV wants to help the people. Accountability lies with the Government and the elected leaders like MCAs and MPs. There is no equity because she is never considered at the public hospital.

**Round Five: Reflection on Respondent 15 (Study site A)**

On this fifth round of our study, we visit our fifteenth respondent and the most important basic service to her is Health because when the babies are not well, they will not come to the center and when the owner is unwell, she cannot run the center. On health, however, my greatest concern here is with the way babies sleep close to each other, coughing over each other thus the high risk of Cross infections and Re-infections of URTIs. These infections transmissions could even affect the owner and her aide because she has fallen sick twice since our second visit and even when she is from hospital, she spends the day at the center.

Hygiene being vital, more involvement is required from the private sector and the government to supplement the current hygiene maintenance efforts done by the community members.

Water is the second most important basic service and its constant inaccessibility points my concern to one dimension of gender that touches on Rights and how despite the universal declaration of human rights, both men and women still have unequal access to basic services like water.

More formal structures must be accountable for Sanitation in the community as people may not be aware of who to turn to legally. The private sector can also play a part in upgrading the Sanitation services within the community.

Both the formal and informal structures that take care of Solid waste must collaborate and come up with a sustainable place for a dump site because the community’s is stuck between a rock and a hard place since they have no place to throw their solid waste.

Since not all day care owners are able to attend beneficial Educational seminars on running day care centers, those organizations facilitating their trainings can opt to go mobile and give the trainings at the day care centers, among other different methodologies.

1. **Health**

As an informal structure, her fellow Day care teacher is the most influential because she handles the day care when our participant is not feeling well. She calls her on her phone and they keep in touch throughout the day. She gets her authority from herself and she wants the babies’ health to improve such that when there is medicine to be taken by the babies, she knows how to administer it.

When the Day care owner goes to the City council public hospital, she will be made to line up even if she explains that there are other babies waiting for her attention at the center. At the private hospital, she got prompt treatment after explaining that there are babies at her day care center waiting for her. Students in uniform get prompt treatment so that they can go back to school, in a public hospital. When a woman and a man go to hospital, both line up and tow the line, but an expectant lady will be given priority. A PLWD will line up at the public hospital because people do not like being cut while on the line, saying that they too have needs. Accountability lies with formal structures like the government to ensure that public hospitals have medicine and the Doctors who vowed to serve the community. There is no equity at the public hospital while at the private hospital, one gets good service because of paying for it and the government is not aware that there is marginalization and discrimination inside the city council public hospital, but the Village elders and the staff can report on such cases. Community members and NGOs can also get that information out.

1. **Hygiene**

As an informal structure, the Day care owner is the most influential because she cleans and maintains the center daily and she wants the babies and children to stay in a good and clean environment, with authority from herself.

Accountability lies with the Daycare owner as an informal structure because it is a business and she wants to get customers. As formal structures, the Assistant Chief and the Ministry of health are responsible for monitoring hygiene in the community. There is equity because they take care of their own hygiene at the center but outside the center there is no equity because people do their cleanliness differently.

1. **Water**

As an informal structure, the vendors are the most influential because they supply water and they can be found within the villages. They get their authority from themselves and they want to make money from their work.

The people at the water point let children and PLWDs fetch water without lining up. Accountability lies with the Day care owner as an informal structure, responsible for fetching water when it is available, to preserve it for the time it is scarce. The government, as a formal structure, is also accountable in ensuring accessibility. There is no equity because water accessibility is not constant.

1. **Sanitation**

The Day care owner is the most influential as an informal structure because she maintains the plot toilet.

The plot toilet is not PLWD friendly, but children who cannot use the toilet use potties.

Accountability lies with the plot Care taker who should tell the new tenants about the plot rules for tenants to be more responsible and she gets her authority from the Landlord. There is no equity because the tenants in the plot do not help her with washing the toilet yet they use it.

**Round Six: Reflection on Respondent 15 (Study site A)**

On this day of our sixth visit to our fifteenth participant, she is not feeling very well yet she agrees to continue with the interview since there are only two baby girls and the autistic boy who earlier hit his head on the wall while playing so we find him a bit quiet, with a sore bruise protruding on his forehead. He cheers up after we enter the room and when the teacher presses ice gently on the sore to cool it, he runs away from her and goes to sit next to my colleague, making us appreciate how he identifies with his gender, with a light touch. Soon afterwards, as we continue with our interview, he goes back to the teacher, who feeds him his water melon as he plays around the room. Sometimes as he approaches me wanting to play and I grab him playfully and hold him down to give the teacher ample time to reflect on my questions and focus on the interview because she gets pre-occupied with him, causing me to pause the interview a few times. He settles down once he has wet his diaper and I pause the interview to let our participant change his diaper, after which we continue, as he lays down next to his her to sleep. She is visibly not in her usual spirits and what works here is the way I mirror back her responses to show her that I am genuinely into our conversation. By the end of our interview, our participant wakes up the other two children and starts to prepare them for home.

The closer of schools has changed the opening times at the center where previously she could open at 6.00 am but now parents bring their children after 7.00 am. It has greatly reduced the number of children going to the center since they are taken care of by their older siblings and it is cheaper for the parents. Without children, she is not able to pay her aide so for the past week, she has been running the center alone with minimal babies. This has brought the numbers of children using the plot toilet to zero and the few left use only two potties, with water consumption reducing by half to 60 liters a day, with most of it going to hygiene maintenance and cooking.

1. **Sanitation**

As informal structures, the most influential are the Tenants because they each have a scheduled day for maintain the plot toilet and they communicate with their Landlord as a formal structure via the phone whereby if need be, he will come to the plot, with authority from the Village elders and the Chief, who together with NGOs, are accountable for proper sanitation in the community. It also lies with Well-wishers and a trusted Tenant to ensure the plot rules are adhered to and the toilet washing rota is followed, including giving updates of the plot toilet condition to the Landlord and the government’s Ministry of Housing and Planning which gives authority to the Village elders and the Chief. The Landlord has interest in reducing the people’s suffering when accessing toilets by ensuring his plot has a toilet. There is no equity because not everyone can access free toilet facilities and it will be achieved when government rules are set such that when one is building a plot, they must reserve space for building toilets. On the other hand, NGOs, through Community groups, have played a part in ensuring equity by building toilets in some villages, and they are found through the community groups that work with them, both getting authority from the Village elders and the Chief. Community groups have interests in helping the community. The toilets in her plot are not PLWDs, small children and elderly friendly because of their high door steps. One public toilet at the Chief’s camp, is PLWDs and gender friendly, thus ensuring equity in the area.

1. **Water**

As informal structures, Community Youths with carts are the most influential as they supply water during shortage, thus reducing marginality. Accountability lies with the Village elder and the area MCA who responds to the people’s complaints, through his agents from the community, set at central points on the ground and he has interest in the whole community accessing water. NCC (Nairobi City Council) is also accountable in filling up water tanks in the community. There is no equity since not all areas have accessible water, even without the shortage. The smaller the water container, the higher the chances of a child in home attire getting priority at the water point, however, marginality is reduced when the community members at the water point respect the elderly enough to give them priority to fetch water at the point.

1. **Hygiene**

As an informal structure, Tenants are the most influential such that for the Day care Owner/teacher, she is the one who maintains the cleanliness of the plot where her center is, because she wants the parents to find the place clean when they bring their babies, and also because the Care taker cleans the front area only, yet there is the inside verandah, kitchen and toilet to be maintained as they are always in use on a daily. Back at home in her plot, it is the tenants who maintain cleanliness daily. Accountability in sensitizing people on hygiene maintenance lies with the Community members, NGOs who get their authority from the Ministry of Health, reachable through the Chief, and who ensure that community members maintain hygiene so as to prevent diseases. There is no equity because some areas are clean and others are not, and it can be achieved when people are trained and sensitized on the effects of poor hygiene and the benefits of high hygiene. Men leave the hygiene responsibility for maintaining cleanliness in the plot. A man finds it hard to clean the open front of the plot area as he imagines the perceptions of the people who will see him do a woman’s work, that will make him feel ashamed, while to a woman, cleaning open areas is a shameless normal thing. Elderlies and PLWDs find it a challenge to maintain hygiene when it involves activities that require frequent bodily movements that require stamina.

1. **Health**

As formal structures, the most influential are the Doctors at the Private hospitals because most parents prefer to take their children there for comprehensive health care. Doctors are interested in treating community members and they get their authority from their organizational bodies that oversee them. Accountability lies with the Government’s Ministry of Health who authorize both private and public bodies. Marginality and vulnerability is reduced when a PLWD and an elderly are given priority at the hospital. Equity is ensured during nationwide vaccination campaigns because they penetrate the villages.
